# GeVaDSs – decision support system for novel Genetic Vaccine development process

**DOI:** 10.1186/1471-2105-13-91

**Published:** 2012-05-10

**Authors:** Jacek Blazewicz, Marcin Borowski, Wahiba Chaara, Pawel Kedziora, David Klatzmann, Piotr Lukasiak, Adrien Six, Pawel Wojciechowski

**Affiliations:** 1 Institute of Computing Science, Poznań University of Technology, 60-965 Poznań, Poland; 2 Institute of Bioorganic Chemistry, Polish Academy of Sciences, Poznań, 61-714, Poland; 3 CNRS, UMR 7211, Immunology, Immunopathology, Immunotherapy, 75013 Paris, France; 4 UPMC Univ Paris 06, UMR 7211, Integrative Immunology team, 75013 Paris, France; 5 AP-HP, Pitié Salpêtrière Hospital, Department of Biotherapy, 75013 Paris, France; 6 INSERM, U959, Immunology, Immunopathology, Immunotherapy, 75013 Paris, France

## Abstract

**Background:**

The lack of a uniform way for qualitative and quantitative evaluation of vaccine candidates under development led us to set up a standardized scheme for vaccine efficacy and safety evaluation. We developed and implemented molecular and immunology methods, and designed support tools for immunization data storage and analyses. Such collection can create a unique opportunity for immunologists to analyse data delivered from their laboratories.

**Results:**

We designed and implemented GeVaDSs (Genetic Vaccine Decision Support system) an interactive system for efficient storage, integration, retrieval and representation of data. Moreover, GeVaDSs allows for relevant association and interpretation of data, and thus for knowledge-based generation of testable hypotheses of vaccine responses.

**Conclusions:**

GeVaDSs has been tested by several laboratories in Europe, and proved its usefulness in vaccine analysis. Case study of its application is presented in the additional files. The system is available at: http://gevads.cs.put.poznan.pl/preview/(login: viewer, password: password).

## Background

### Immune response & vaccination principles

Vaccines are effective tools to prevent infectious diseases in humans or animals [1,2]. A vaccine typically contains an antigen mixture that stimulates a body’s immune system to recognize the agent as foreign, inducing specific immune responses and memory for long-term protection against the disease-causing microorganisms. Vaccines can be prophylactic (to prevent a future infection) or therapeutic (e.g. vaccines against cancer). Following vaccination, both innate and adaptive immune system components synergize to elicit an immune response. Antigen-presenting cells - notably dendritic cells - take up antigens and traffic to the draining lymph nodes where they present processed antigens to naive CD4+ and CD8+ T lymphocytes. Naive T-cells are stimulated to proliferate and differentiate into effector and memory T-cells. Activated effector and memory CD4+ T-cells provide help to B-cells to mount antibody responses, and help naive CD8+ T-cells to enhance their clonal expansion and differentiation into cytotoxic CD8+ T lymphocytes (CTL). The quality of the vaccine-induced immune response depends on several factors, e.g. antigen nature, route of administration, antigen presentation, vaccine preparation adjuvants, or timing between challenges.

### Vaccine development & evaluation

Usually, the effectiveness of vaccination is ascertained when vaccinated individuals exposed to infection are protected. To be effective, a vaccine should trigger efficient activation of antigen-presenting cells to initiate antigen processing and presentation to T-cells, as well as activation of T and B-cells (production of antibodies, generation of memory B-cells, memory CTL and memory CD4+ T-cells). Several immune parameters can be measured to monitor the immune response, including antigen-specific and neutralizing antibodies, antigen-specific T-cell expansion, CTL activity and cytokine production. Dendritic cell state of activation can be assessed by transcriptome analysis. A central goal of vaccine research is to identify whether an early vaccine-induced immune response is predictive of later protection [3,4]. An immune response can be used for guiding vaccine development, for predicting vaccine efficacy in different settings, and for setting vaccination policies and regulations.

The new generations of recombinant vaccines comprise peptides, DNA vaccines, viral or bacterial delivery system and Virus Like Particles. Although enormous progress has been made in the process of vaccine development, there is still a need to standardize the qualitative and quantitative evaluation of new vaccines, to develop a platform of novel recombinant genetic vaccines using genomic and proteomic information and to create interactive systems to store all types of experimental data together (such as immune response parameters stated above) with statistical and analytical tools to support scientists during their experiment. The above goals were achieved by the CompuVac consortium [5] established within a project funded under FP6 EC programme. As one of the key deliverable of this project, a new web platform called GeVaDSs (Genetic Vaccine Decision Support system) has been designed and implemented. GeVaDSs is accessible for scientific community. GeVaDSs allows one to (i) find the description of stored experiments, (ii) create new experiments and store experimental data, and (iii) visualize and cross-compare the results of theses experiments. The construction and the development of the GeVaDSs is a first trial to standardize and describe the whole process of vaccine development, making it a really challenging aspect of the CompuVac project.

Although it is difficult to find efficient system for vaccine analysis, it is worth to mention results of VIOLIN initiative (Vaccine Investigation and Online Information Network) [6,7]. It is a system for storing and analyzing published vaccine data. It has been developed for data mining, curation and analysis of results for commercially available vaccines, as well as vaccine candidates on the early stage of development. The main difference between VIOLIN and the one presented in GeVaDSs is the level of data collected in the system and the analysis possible. The first system gives a researcher an opportunity to gather data concerning a particular vaccine. The second one is useful at the level of laboratory tests of those vaccine. GeVaDSs was developed to store raw experimental results and presents them in a user friendly form. It allows for a comparison of the organism response to a vaccination at different levels like T-cell and B-cell. Furthermore, the influence of the vaccine on the selected gene expression can be determined in the *Molecular Signature* module and the efficacy of the immunization protocol used, is analyzed in the *Vector Challenge* module.

## Implementation

The system was implemented using the Adobe Flex 3 technology and Action Script 3 language on the client side (interface, logic mechanisms), whereas the server side interface was implemented using mainly the PHP script language, in addition to other languages like Perl, R and Java. All data processed by the GeVaDS system are stored in the PostgreSQL relational database.

### Structure of the system

**Figure 1:**
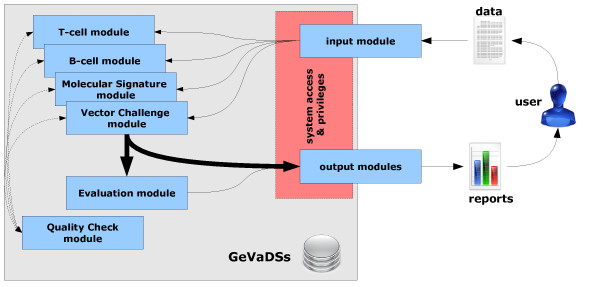
**The structure of the GeVaDS system.** The simplified structure of the GeVaDS system with main modules and connections among them.

GeVADSs consists of two groups of modules: *vaccine-related modules*, which are designed to process the biological data, and *support modules*, which are responsible for data entry, report generation and data access. The simplified structure of the system is presented in Figure 1.

The vaccine-related modules are: *T-cell*, *B-cell*, *Molecular Signature*, *Vector Challenge*, *Evaluation* and *Dictionaries* (the latter is shared by all other modules and is not shown in Figure 1). Their brief descriptions are presented below. The support modules are: *input* for introducing data, *output* for viewing the results, *Quality Check* - for checking the quality of introduced data, reports which allows the user to generate a report for requested data as a pdf file, and the system access and privileges. All of the support modules, excluding the last one, are designed separately for each of the vaccine-related modules. For example, GeVaDSs consists of *T-cell input* module, *B-cell input* module etc. Obviously, input modules are very similar.

#### Dictionaries

As mentioned above, *Dictionaries* is a general module, which is shared by all modules to unify terms and definitions used in experiments definition. It defines basic terms like species, animal models, antigens... used across the whole system. However, some parts are crucial to understand the idea of GeVaDSs, and therefore are presented below in more details.

##### Hierarchy of vaccine vectors

**Figure 2:**
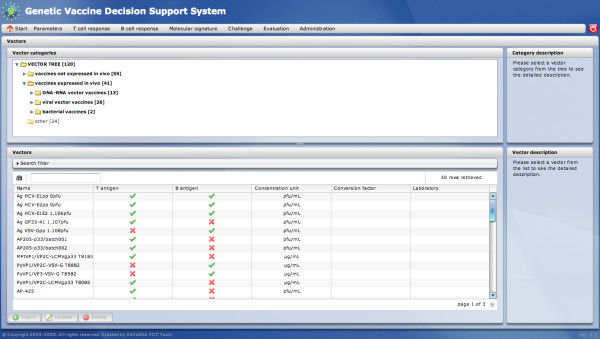
***Vector *module - main view.** The *Vector* module main view presents the tree of vector categories and a list of vectors in a selected position of the vector category subtree.

Hierarchy of vaccine vectors has been created on the basis of their biological features. Each vector in the hierarchy has a standardized description which contains information about its producer, production type, antigen, the expected type of immunological response, etc. The standardization of vectors enables their evaluation based on the quality of vaccine-induced immune responses. The GeVaDS system not only provides a set of defined vectors arranged in a hierarchy, but also enables a user to store and manage his own vectors and put them into the hierarchy. The *Vectors* module makes it possible to edit information about vectors. The main view of this module is divided into two main parts: the tree of vector categories and a list of vectors in a selected position of the vector category subtree (Figure 2).

##### Immunization protocol

**Figure 3:**
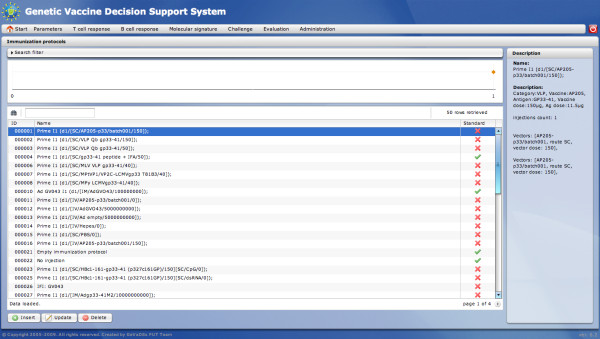
***Immunization protocol *module - main view.** The *Immunization protocol* main view presents the list of defined immunization protocols and the time chart representing defined inoculations.

The *Immunization protocol* module has been designed to standardize and store data about immunization protocols and to define immunization process. An immunization protocol is created by a careful description of each inoculation stage (schedule, vector, dose, administration route). Hence, each immunization protocol is standardized in the system. Once a protocol has been created, it can be displayed in the form of a time chart, on which timepoints of inoculations are represented. Each of these timepoints has a detailed description presented as a tooltip (displayed on the mouse-over action). The list of defined immunization protocols is presented on the grid with the most significant parameters displayed in columns. A search filter filters immunization protocol entries based on user demands. The main view of this module is presented in Figure 3.

##### Challenge protocol

**Figure 4:**
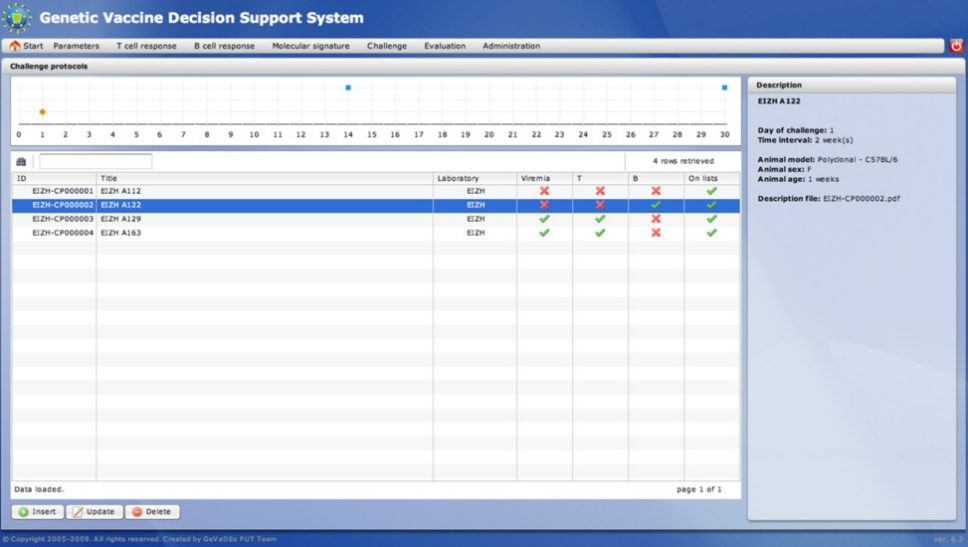
***Challenge protocol *module - main view.** The *Challenge protocol* main view presents the list of defined challenge protocols and the time chart displaying defined dates at which the animal survival is measured.

The *Challenge protocol* module has been designed to define and store information about challenge protocols used to evaluate the susceptibility to an infectious challenge with a pathogen of a population of individuals, vaccinated or not. A challenge protocol is created by defining information provided about the challenge, i.e. animal description, dates or intervals of viremia, T-cell response and/or B-cell response. Hence, each challenge protocol is standardized in the system. Protocols already defined can be displayed as time charts. The meaning of particular actions is the same as in the case of the immunization protocol. The main view of this module is presented in Figure 4.

##### Organization of experiments

*Experiment* is the basic assessment unit in the system. It describes a vaccination experiment conducted in the laboratory, and stores data such as: information about experiment creator, date, default parameters of the experiment (default animal model, default sex, default age...) which would be inherited by the experimental group unless otherwise specified. Default parameters are thus helpful when many experimental groups are similar to each other only with slight differences. Each experiment introduced into the system involves several *experimental groups*. An experimental group consist of a set of uniform individuals (same age, same sex, same animal model...) for which the immunization followed a given protocol.

There are five types of experimental group. Each experimental group can be one of the following types: 

· experimental vaccine - a group of individuals being immunized with an experimental vaccine to be tested.

· naive/control - a group immunized with an empty vector, without antigen.

· negative control - no immunization.

· Internal Standard - a group being immunized using a predetermined standard immunization protocol based on the selected internal standards, which constitutes a benchmark for comparison of different experiments (e.g. carried out in different laboratories).

**Figure 5:**
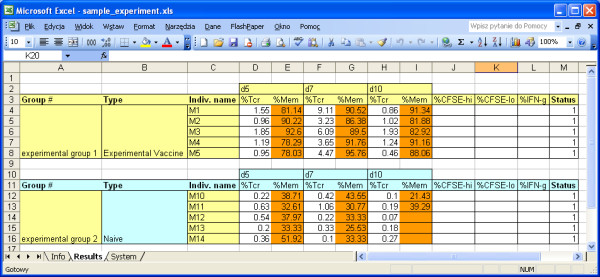
**Example of experimental data template.** The template provides a very easy way of introducing all results of an experiment to the system by simply copying and pasting results from other files, and then importing the template into the system.

The system enables to generate an Excel template of experiment (Figure 5) after introducing into the system very basic information to define the experiment. The template provides a very convenient way of introducing all results of an experiment to the system by simply copying and pasting results from other files, and then importing the template into the system. Templates constitutes parts of the *input modules*.

##### System access & privileges

The usage of the GeVaDS system requires authorization. This mechanism not only protects the system against accidental usage or misbehavior, but also ensures safety of data stored in the system. Authorized users have access to modules depending on the role assigned by the system administrator (e.g. *admin*, *lab manager*, etc). Privileges defined in the system are divided into two groups: those connected with the access to particular modules or with performing some actions, and those connected with the user data. Privileges for the first group could be assigned by the system administrator (they are called *Roles*). The second type of privileges are managed by users themselves. Each user has a full access to his/her own data (as the *owner* of the data), a read-only access to public data and access to data shared by other users. The *owners* can make their data publicly accessible or they can give a custom access to other users. However, by default, the *lab manager* (a special *role*) of a given laboratory has full access to all data of users belonging to his/her laboratory.

### Module description

#### Quality Control module

*Quality Control* module is responsible for checking the quality and correctness of the data introduced by the user as well as the composition of experiments. The quality control process is based on a set of quality rules. Each rule is assigned a unique number, information about the data module to which the rule should be applied (*T-cell, B-cell, Molecular Signature* etc.) and the level of application (experiment, experimental group, individual or data value). Depending on the level of application, there are rules responsible for checking the definition of experiments or experimental groups (i.e.: Have the laboratory & date of experiment been provided? Have experimental control groups been created? Is each experimental group large enough for statistical analysis?...), as well as controlling every single value of all attributes (i.e.: Are interferon gamma production values provided as percentages? Are the naive experimental group results in acceptable ranges?...). Reference values of attributes defining acceptable ranges have been defined by immunology experts.

The procedure of quality control of an experiment (or an experimental group) should be launched by the user before data analysis. During this procedure, in case a rule is broken the system generates an error message. At the end of the procedure a report listing all messages is produced. Error messages belong to the followings categories: 

· warning

· minor error

· serious error

· fatal error

**Figure 6:**
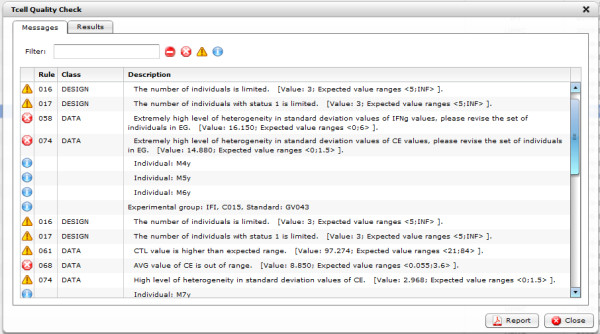
**Quality control report - list of messages.** An example list of messages of an experiment quality control report.

Each error is marked with an icon in the first column of the report (see Figure 6).

After the quality control procedure, each experiment, as well as experimental group, receives a *quality status*. Five different quality statuses have been defined: 

· *Not Checked (NC)* - the default status before launching the quality control procedure. Quality status is reset to NC after any revision.

· *Rejected (R)* - the data for experimental group or experiment have some fatal errors and therefore the data need to be corrected before proceeding.

· *Pending (P)* - the data for experimental group or experiment have some serious errors; the user may decide to manually enforce the quality status from Pending to Rejected or User.

· *User (U)* - the data for experimental group or experiment have been manually validated by the user (see Pending).

· *Auto (A)* - the data for experimental group or experiment have no fatal nor serious errors and have been automatically validated by the system.

**Figure 7:**
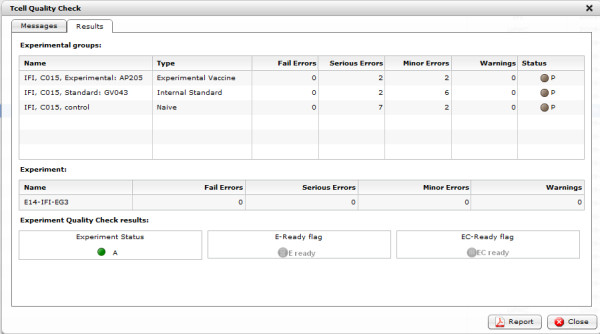
**Quality control report - summary.** An example summary of an experiment quality control.

Experiments or experimental groups with the status Auto or User are assumed to be valid and ready for further analysis. Figure 7 shows an example summary report.

Each experiment may also have one of two quality flags: 

· *E-ready* - means that the experiment has reliable data and involves correct experimental groups that can be analyzed. The *E-ready* flag is received when at least one Naive and one Internal Standard or Experimental Vaccine groups have the Auto or User quality status.

· *EC-ready* - means that data stored for this experiment can be analyzed and compared against other experiments. The *EC-ready* flag is received when at least one Naive, one Internal Standard, and one Experimental Vaccine groups have the Auto or User quality status.

#### T-cell module

*T-cell* module analyzes the response mediated by T lymphocytes over time. This response is described by the following parameters: expansion of vaccine antigen-specific T-cells (%), memory phenotype (%), CTL activation (% of intracellular interferon gamma positive CD8^+^T-cells) and cytotoxicity (% of specific lysis).

T-cell response values can be introduced in two ways: *manually*, by introducing values using the *T-cell input* submodule or *automatically*, by generating and uploading a standardized template (a Microsoft Excel file) that can be filled by the user by copy-paste from his/her worksheet and then uploaded back into the system. After checking for consistency, data is automatically inserted and quality-controlled.

These alternative input methods raise the problem of a possible conflict between automatic uploading of a template and manual edition of the experiment after template generation. This problem has been circumvented by stamping both the template and experiment at the time of template generation, the experiment stamp being modified during any manual edition. Therefore, in the latter case, an older template version will be rejected for uploading.

**Figure 8:**
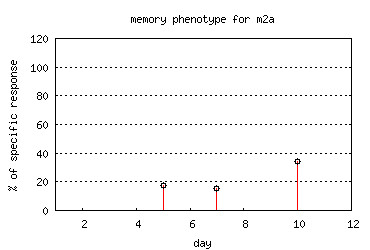
***T-cell response *module - individual chart.** An example chart taken from *Individual* submodule of *T-cell response* module representing the change of memory phenotype over time.

**Figure 9:**
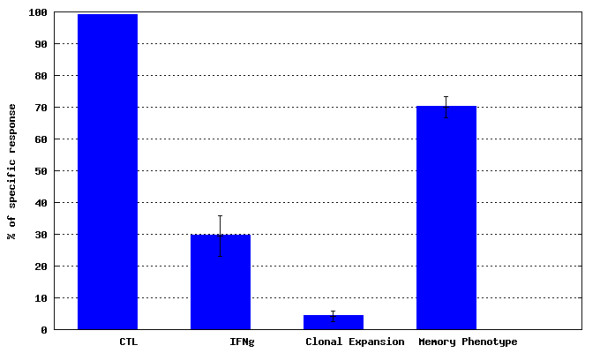
***T-cell response *module - average results for an experimental group.** An example chart taken from *Experimental group* submodule of *T-cell response* module representing average values for T-cell response attributes.

**Figure 10:**
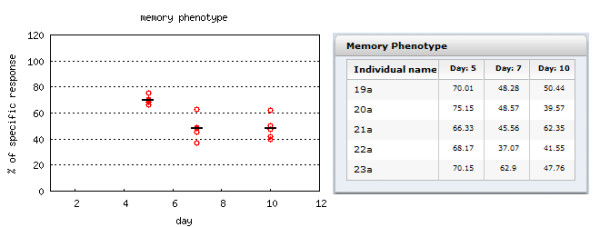
***T-cell response *module - summary of individual values for an experimental group.** An example chart and data table taken from *Experimental group* submodule of *T-cell response* module representing values for individuals of a selected experimental group.

**Figure 11:**
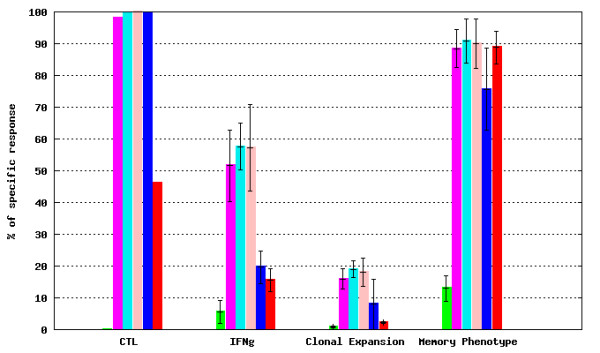
***T-cell response *module - experimental values from a selected experiment.** An example bar chart taken from *Experiment* submodule of *T-cell response* module representing average values for experimental groups (after normalization against naive experimental group data). See GeVaDSs Manual for detailed description of calculation [14].

Once data have been properly uploaded and quality-controlled, they can be analyzed with T-cell response submodules: 

· *Individual* submodule, where data for individuals can be displayed as charts and tables. Figure 8 shows an example chart for a given individual,

· *Experimental group* submodule, where data for all individuals within an experimental group can be analyzed. This submodule displays charts and tables for average (Figure 9) and individual (Figure 10) values for the selected experimental group,

· *Experiment* submodule, where data for experimental groups can be compared. In order to facilitate result evaluation, this submodule offers several graphical and tabular outputs, e.g. bar charts (Figure 11), radar charts (not shown).

**Figure 12:**
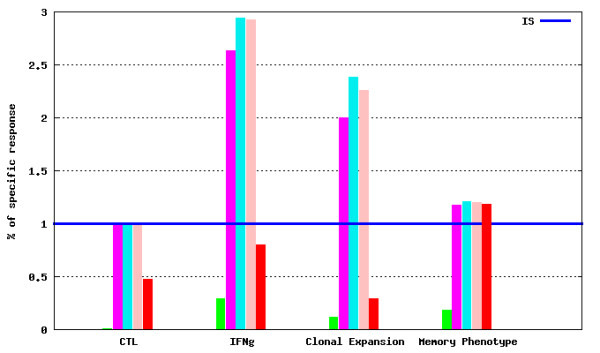
***T-cell response *module - experimental values from a selected experiment compared to the Internal Standard.** An example bar chart representing experimental group results normalized against Internal Standard experimental group results (displayed as the Internal Standard reference line).

**Figure 13:**
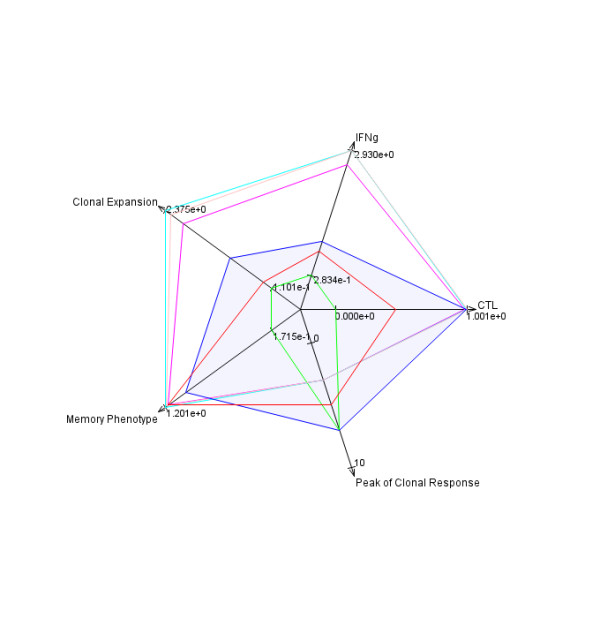
***T-cell response *module - radar chart for a selected experiment in comparison to the Internal Standard.** An example radar chart representing experimental group results normalized against Internal Standard experimental group results (see Internal Standard reference greyed area). The axis of the chart are oriented in the way that the best values for a particular parameter are positioned outside. Therefore, the better results for an experimental group the bigger area covered by the polygon for this group on a radar chart.

· *Comparison of Experiment* submodule where data for experimental groups belonging to different experiments also can be compared. Such a comparison is possible when the experimental groups are related to the Internal Standard groups immunized following the same Immunization Protocol. Results of selected experimental groups are presented as ratios against the selected Internal Standard group results. Example bar and radar charts are presented in Figures 12 and 13, respectively.

#### B-cell module

*B-cell response* module analyzes the response mediated by B lymphocytes in terms of antibody production and antibody neutralization activity. The results are presented as sigmoid curves obtained after linear regression for serum titration [8]. Since calculating the sigmoid is computationally time consuming, the system calculates the sigmoid once on first request. Linear regression results are stored in the database.

For each experiment, the following parameters should be defined: 

· serum descriptions (typically marked as S0, S1...) - which are defined for different days of measurement,

· serum dilutions (marked as 1/20, 1/100...) - which are defined for different dilutions tested.

The structure of the *B-cell response* module is very similar to that of the *T-cell response* module. The main difference concerns the data insertion requirement since the definition of the experiment is a prerequisite step before template creation, as well as the definition of serum descriptions and dilutions. Once experiment is properly described, the B-cell template is found very convenient with regard to the much higher amount of data to be provided for titration experiments.

**Figure 14:**
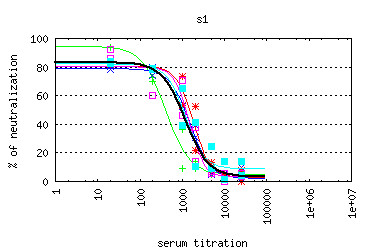
***B-cell response *module - sigmoid chart.** An example sigmoid chart taken from *B-cell response* module. The chart shows the sigmoids calculated for several individuals for S1 serum titration.

The sigmoid titration curve is expressed as follows: 

· with the sigmoid formula (Eq. 1) [8],

· with a table with the sigmoid parameters values (not shown),

· with a graphical representation of the sigmoid (Figure 14).

$$\begin{array}{cc} \mathrm{\%neutralization}(\mathrm{dilution}) & =\mathrm{bottom}\\ \;+\frac{\mathrm{top}-\mathrm{bottom}}{1+10^{\mathrm{HillSlope}*\mathrm{lo}g_{10}\left(\frac{\text{EC}50}{\mathrm{dilution}}\right)}}, \end{array}$$

where: 

· *top* - is a maximal curve asymptote,

· *bottom* - is a minimal curve asymptote,

· *HillSlope*- denotes the steepness of the curve,

· *EC*50 - denotes the dilution that produces the neutralization halfway between the *bottom* and *top* response levels.

Again, several output submodules can be distinguished in the *B-cell* module.

**Figure 15:**
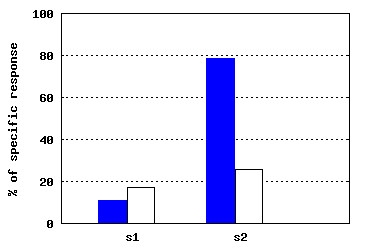
***B-cell response *module - neutralization chart.** The chart shows neutralization results across dilutions for Ag-specific pseudoparticles (Ag-pp - blue color) and Control Ag-irrelevant pseudoparticles (CTR-pp - white color). Charts are taken from the *Individual* submodule of *B-cell response* module.

*Individual* submodule allows the user to analyze the sigmoid for a single individual. Charts display the neutralization per dilutions for Ag-specific pseudoparticles (Ag-pp) and Control Ag-irrelevant control pseudoparticle (CTR-pp) (Figure 15).

*Experimental group* submodule shows the sigmoid for all individuals assigned to the selected group, and the average sigmoid (determined by a linear regression for all data points). The results also contain the set of charts with the neutralization per dilutions for Ag-pp and CTR-pp together with the average values of dilutions.

**Figure 16:**
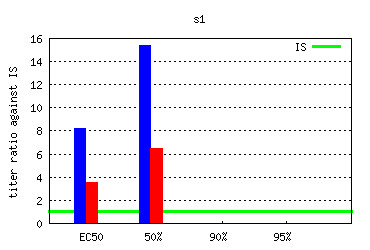
***B-cell response *module - sigmoid-based antibody titer chart.** An example chart taken from the *Experimental group* submodule presenting the antibody titer values deduced from sigmoid regression for selected individuals. See GeVaDSs Manual for detailed description of calculation [14].

*Experiment* submodule shows the outcome in the same way as the Experimental group submodule but the data for sigmoid calculations include all data points for all individuals for each experimental group within the selected experiment. When selecting the Internal Standard, an additional series of charts (Figure 16) are presented after normalization against the Internal Standard group.

**Figure 17:**
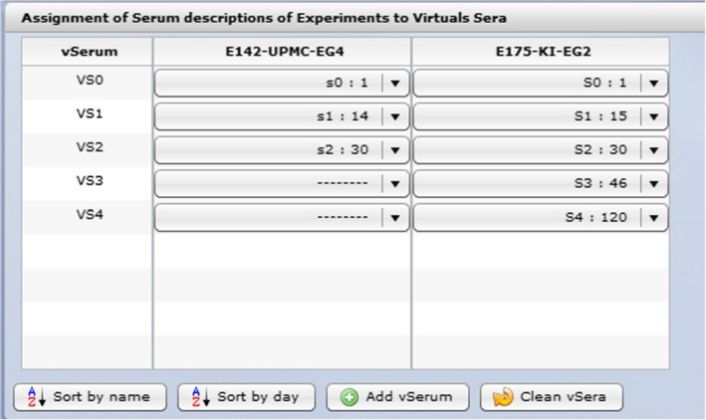
***B-cell response *module - serum correspondence editor in *Comparison of experiment *sub-module.** In case of a comparison across experiments, the problem is that different titration days can be specified in different experiments. Therefore, the system cannot automatically compare the data. The term *Virtual Serum* was introduced by Compuvac team to solve this dilemma. In the *B-cell response* module, a user specifies which results should be compared. In other words, when the comparison is between experimental groups belonging to the same experiment, the charts present results for each of the serum titration separately. When the user compares results of experimental groups from different experiments, the charts are created for each of the Virtual Sera assigned. In the provided example, two experiments with different numbers of serum descriptions taken at different days (see VS1) are compared. This “Virtual Serum” editor allows to match data of the 14’th day of experiment *E142-UPMC- EG4* with the 15’th day of experiment *E175-KI-EG2*.

*B-cell response* submodule allows to analyze results for experimental groups across experiments by experiment comparison functionality. This submodule offers the possibility to evaluate, automatically or manually, sera across experimental groups. An example is provided in Figure 17.

The outcome provides normalized sigmoids for all selected experimental groups, quality charts and data tables.

#### Molecular Signature module

The main purpose of the *Molecular Signature* module is to extract a gene subset, the expression of which varies after immunization. Such gene signatures could then be used as markers of vaccine efficacy. *Molecular Signature* module characterizes the change of gene expression of vaccinated individuals against that of control individuals. Input data for this module are results of microarray experiments which are introduced into the system. The organization of the experiment data is similar to that in the other modules. Hence, a user defines an experiment, experimental groups, and individuals within each experimental group. For each individual, microarray results can be introduced for various organ and cell types. Currently, the system is able to analyze Illumina, CodeLink & Affymetrix microarray data. Each experiment, as well as experimental group, has a quality status which defines the quality of the data (see Quality Control module section).

Gene expression analysis can be restricted to predefined gene lists of interest, created and managed by users.

**Figure 18:**
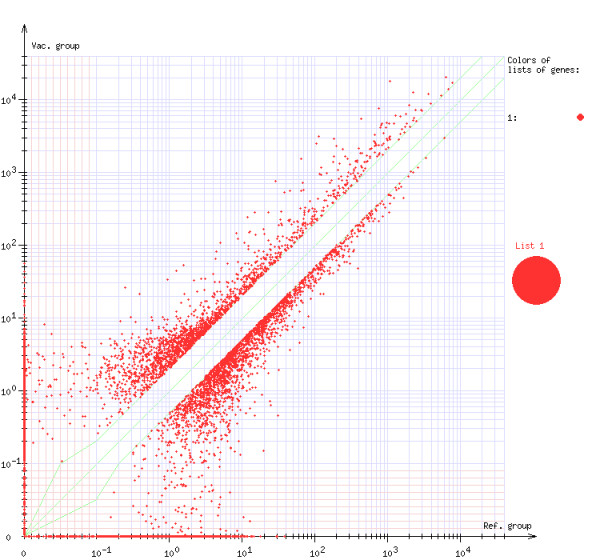
***Molecular signature *module - comparison of gene expression value.** The Figure presents a comparison of gene expression of a vaccinated group of individuals against the control group. A spot represents a gene. Position of a spot is determined by the expression of a given gene. Vertical position of a spot represents expression of the gene of the vaccinated group while horizontal position of a spot represents expression of the gene of the control group. Filtering mechanism could be applied in order to remove from the analysis genes, which expression’s ratio is lower than some threshold (the empty space on the diagonal of the chart).

**Figure 19:**
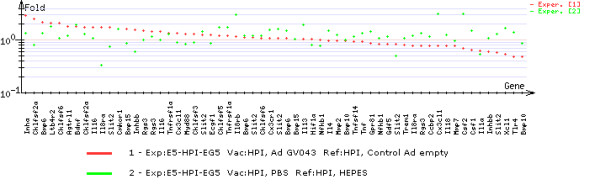
***Molecular signature *module - comparison of gene expression changes.** Presents the comparison of gene expression changes of several experiments simultaneously.

The analysis of microarray data can be done in *Results* and *Comparison of experiments* submodules. *Results* submodule presents the comparison of gene expression of a vaccinated group of individuals against the control group, using graphical format (Figure 18). *Comparison of experiments* submodule presents the comparison of gene expression changes in several experiments simultaneously (Figure 19).

#### Vector Challenge module

*Vector Challenge* module measures the efficacy of immunization protocols. It stores survival, or protection, information of vaccinated individuals challenged with live virus (against which the vaccine, if efficient, is expected to protect the individual).

Again, *Vector Challenge* result parameter values can be inserted by generating an MS Excel template file after defining necessary information about the experiment, experimental groups and number of individuals within each group. The template can be filled by the user by copy-pasting from his/her worksheet and then uploading back to the system. After checking for potential inconsistencies, uploaded data will be inserted automatically. In case of errors, a report is generated. The mechanism of template generations and template uploading is similar to that in *T-cell* and *B-cell* modules.

The following data analysis submodules have been developed: 

· *Individual* submodule, which allows the user to analyze single individuals. Data can be presented in a tabular way and as a chart.

· *Experimental group* submodule, which allows the user to view an outcome of challenge results for a selected experimental group presenting the average values for individuals. Results are presented in a chart as well as a data table (not shown).

**Figure 20:**
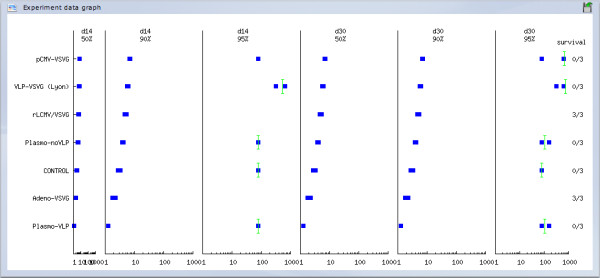
***Challenge response *module.** An exemplary data graph taken from *Challenge response* module. For each vector tested (as indicated on the left), antibody titers (measured at days 14 and 30) and individual survival (as indicated on the right), are summarized.

· *Experiment* submodule, which allows the user to view the outcome at the experiment level. Results are presented in several useful formats using tables and charts (Figure 20).

#### Evaluation module

The idea and implementation of the *Evaluation* module is a unique solution in the current state of the art of immunological experiments. The idea that lies behind this module is to assess experiments using multicriteria analysis. *Evaluation* module provides an overall evaluation of investigated vaccines and vectors. The evaluation process establishes effectiveness of a vaccine, based on the results received for the monitored parameters for all types of experiments (*T-cell response, B-cell response, Molecular Signature*).

**Figure 21:**
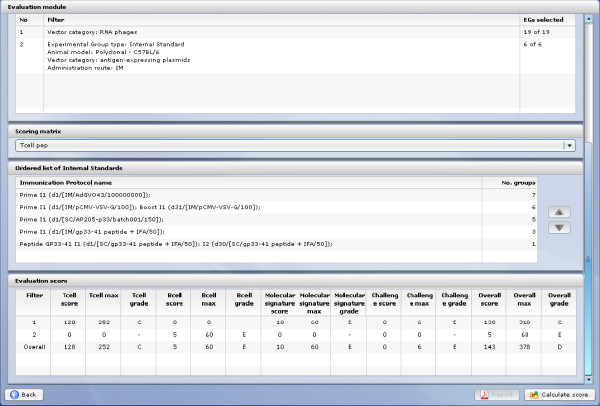
**Evaluation score.** As a result of experiment comparison user can obtain separate scores for each particular experiment, together with the global score.

Each type of experiment has its own weight (it can be adjusted by a user) and as a result of experiment comparison, the user can obtain separate scores for each particular experiment, together with the global score (see Figure 21). Multicriterial analysis can be applied by selecting experiments that should be considered and by setting values of weights corresponding to particular experiment. The quality of experiments is graded from A (the highest) to F (the lowest). This matrix-based analysis provides a standardized frame for comparing vaccine candidates and for identifying most promising results before moving to clinical trials.

## Result and discussion

### Case study

An end-user demonstration is provided as Additional file 1 in order to give a practical tutorial for GeVaDSs main features. The data used for this demonstration example are based on current experimental data produced during the course of the CompuVac project; they are available in GeVaDSs for demonstration purpose so that the corresponding analyses can be reproduced. This simulated example of GeVaDSs illustrates how to test the potential strength of a newly isolated virus (i.e. PEV) as a new vaccine platform by: (i) evaluating the potential of this vaccine platform candidate and (ii) comparing it to other available vector platforms already tested in GeVaDSs. First, the three vector constructs to be tested (PEVdelta, PEVdelta-gp33, PEVdelta-VSVG) are defined in GeVaDSs (slides 3-7). Mice are then immunized with these constructs or CompuVac reference vector (Internal Standard) (slides 8-12). The corresponding T-cell & B-cell immune responses as well as transcriptome data are collected and automatically entered in GeVaDSs database (slides 13-18). Finally, the appropriate analysis reports are generated in order to evaluate PEV vector performance (slides 19-24). As a result PEV is positively evaluated as an efficient vector platform (slide 25) (see Additional file 1). The diagram illustrating the phases of data processing is presented in the chart - see Additional file 2.

## Conclusions

GeVaDSs, developed by the CompuVac consortium, offers a unique solution for improved development of novel Vaccines by the ability to analyse and compare experimental data from different laboratories. The interactive database contains standardized data related to a series of recombinant vaccines (>100) covering most categories of currently developed vaccine platforms (peptide, DNA, viral-based, bacterial-based and VLPs). This data set may serve as a core reference for vaccine developers. The use of the CompuVac Internal standards allows for newly generated data to be added and reliably compared with the core database. During the CompuVac project timeframe, GeVaDSs has already allowed users to conduct comparisons between different vaccine types and initiated novel vaccine design and vaccination regimens. For example, GeVaDSs identified an adenovirus/DNA prime-boost immunization scheme as more efficient than the conventional DNA/adeno prime-boost scheme for vaccination against hepatitis C virus [9]. Besides monitoring of T- and B-cell immune responses, vaccine efficacy and safety profiles were also analyzed by transcriptome studies which allowed the system to generate molecular signatures of different vectors, and their clustering in vector classes. Detailed analyses of these signatures is underway to decipher the underlying networks/pathways correlating with vector activity (manuscript in preparation). We believe that GeVaDSs, whose database is constantly growing, should contribute to rationalizing and speeding up vaccine development.

GeVaDSs has afforded integration of biological and bioinformatics components of immunological experiments. Together with other similar initiatives [7,10], the system provides valuable evidence of the progress in this area and delivers user friendly interface and tools for future analysis of experiments. Recent successful systems biology approaches in the field of immune/vaccine response analysis advocate for larger initiatives and development of standardized databases [11-13]. The flexibility of GeVaDSs makes it possible to adapt the system in a wider field of usage than that specific to CompuVac. This might be of value for acquiring and analyzing datasets associated with translational research investigation in medicine, where multiple parameters are considered with increasing potential for integration of transcriptome, proteome and other datasets, requiring sophisticated bioinformatics-driven analysis, but overlap with more conventional clinical measures. In particular, GeVaDSs is able to assess quantitative measures as employed in clinical trial assays for validation concerning reproducibility, inter-assay and inter-lab variation. GeVaDSs therefore should contribute to shifting traditional and empirical vaccine development to systems vaccinology [3].

## Availability and requirements

· **Project name**: GeVaDSs

· **Project home page**: http://gevads.cs.put.poznan.pl/,

· **Trial version open to the public with read-only rights**: http://gevads.cs.put.poznan.pl/preview/(login: viewer, password: password).

· **Operating system**: Linux for server side

· **Programming language**: client side - Action Script 3.0 and Flex, server side - PHP, c/c++ with GNU Scientific Library, Perl and R.

· **Other requirements**: client side - web browser with Flash Player v. 9.0 or newer, server side - PostgreSQL, Apache 2.0

· **Any restrictions to use by non-academics**: none

The full version of GeVaDSs is publicly available and any researcher can use the system for free after registration. However, the source code of the system is not publicly available and there is no distributable version because of the complexity of implemented modules. The system consists of many connected modules written in many programming languages and installing it is very complicated. In special cases, we are ready to consider an installation of the system for an interested user on his dedicated server.

## Competing interests

The authors declare that they have no competing interests.

## Author’s contributions

DK was the main coordinator of the COMPUVAC FP6 EU project. DK and AS proposed the idea of GeVaDS, whereas JB was a head of the group which was responsible for its creation and guided the final preparation of the paper. AS specified system requirements from biological point of view. PL was a manager of the GeVaDSs’ programmers group. MB, PK and PW designed the system architecture and implemented the whole system. AS, WC were responsible for data curation. WC, AS, PL, MB, PK, PW and others participated in many discussions about the outlook of GeVaDSs modules, what was necessary in this multidisciplinary project. All authors were involved in writing the manuscript and all of them read and approved this version.

## Supplementary Material

Additional file 1 **A simulated example of GeVaDSs use.** Testing the potential of a newly isolated virus as a new vaccine platform. Click here for file

Additional file 2 **GeVaDSs workflow.** The different steps of the GeVaDSs workflow are summarized in the light of the example provided in Additional file 1. Click here for file
