# Supplementary material for: GeVaDSs – decision support system for novel Genetic Vaccine development process
Source: BMC Bioinformatics. 2012 May 10;13:91. doi: 10.1186/1471-2105-13-91 (PMC3531312; doi:10.1186/1471-2105-13-91)
Supplement: Additional file 1 — A simulated example of GeVaDSs use. Testing the potential of a newly isolated virus as a new vaccine platform. [file 1471-2105-13-91-S1.pdf]

A simulated example of GeVaDSs use:

- **testing the potential of a newly isolated virus as a new vaccine platform**

Compuvac warning: This end-user demonstration is provided as a tutorial for GeVaDSs main features. All data used in this example are available in GeVaDSs for demonstration purpose. Although based on actual experimental data produced during the course of the Compuvac project, all characters and facts in this story are purely factitious. Any resemblance with existing characters or facts would be a coincidence…

A simulated example of GeVaDSs use (1)

- Paul, a leading expert in virology, sums up his recent discovery of PEV:
  - *PEV (Pig Encephalitis Virus) was isolated during an epidemic outbreak of encephalitis in pigs in South-East Asia.*
  - *+ss RNA replication in the cytoplasm (no integration in host genome).*
  - *PEV can infect human as well as non-human primate and rodent cells, in particular dendritic cells.*
  - *It can be engineered as non-replicating particles.*
  - *PEV can accommodate 5kb foreign genes.*

- Paul wonders about the potential of PEV as a new vaccine vector :

  → *How to evaluate the potential of this vaccine platform candidate?*

  → *How does it compare to other available vector platforms?*

  → *What antigen to choose and what immune parameters to follow?*

  → *Is there a standardized protocol to evaluate new vectors?*

A simulated example of GeVaDSs use (2)

- Paul searches for “genetic vaccine standardization” and reads about the CompuVac’s strategy and GeVaDSs database interface.
- Paul contacts CompuVac’s coordinator and receives a login to GeVaDSs
- After browsing GeVaDSs user manual and retrieving Compuvac SOPs, Paul decides to proceed and follow CompuVac’s vector testing procedure:

1. Appropriate vector constructs are produced including standard T-cell (LCMV p33-41) and B-cell (VSV-G protein) antigens.
2. The three vector constructs (PEVdelta, PEVdelta-gp33, PEVdelta-VSVG) are added to GeVaDSs vector database.
3. Mice are immunized with vector constructs according to fully documented and standardized immunization protocols. Considering the natural mucosal infection route of PEV, Paul decides to immunize mice intranasally (IN) with 10^8 virus particles.
4. The immunization protocols are added to GeVaDSs.
5. Experimental vaccines are evaluated against controls (naïve animals) and CompuVac reference vector (internal standard).
6. T-cell & B-cell data as well as molecular signature data can be collected and automatically entered in GeVaDSs database (see Exp. E140, E141, E225, E142, E227). Experimental groups are linked to the corresponding immunization protocols.
7. Experiment reports can be generated for vector performance evaluation.

A simulated example of GeVaDSs use (3)

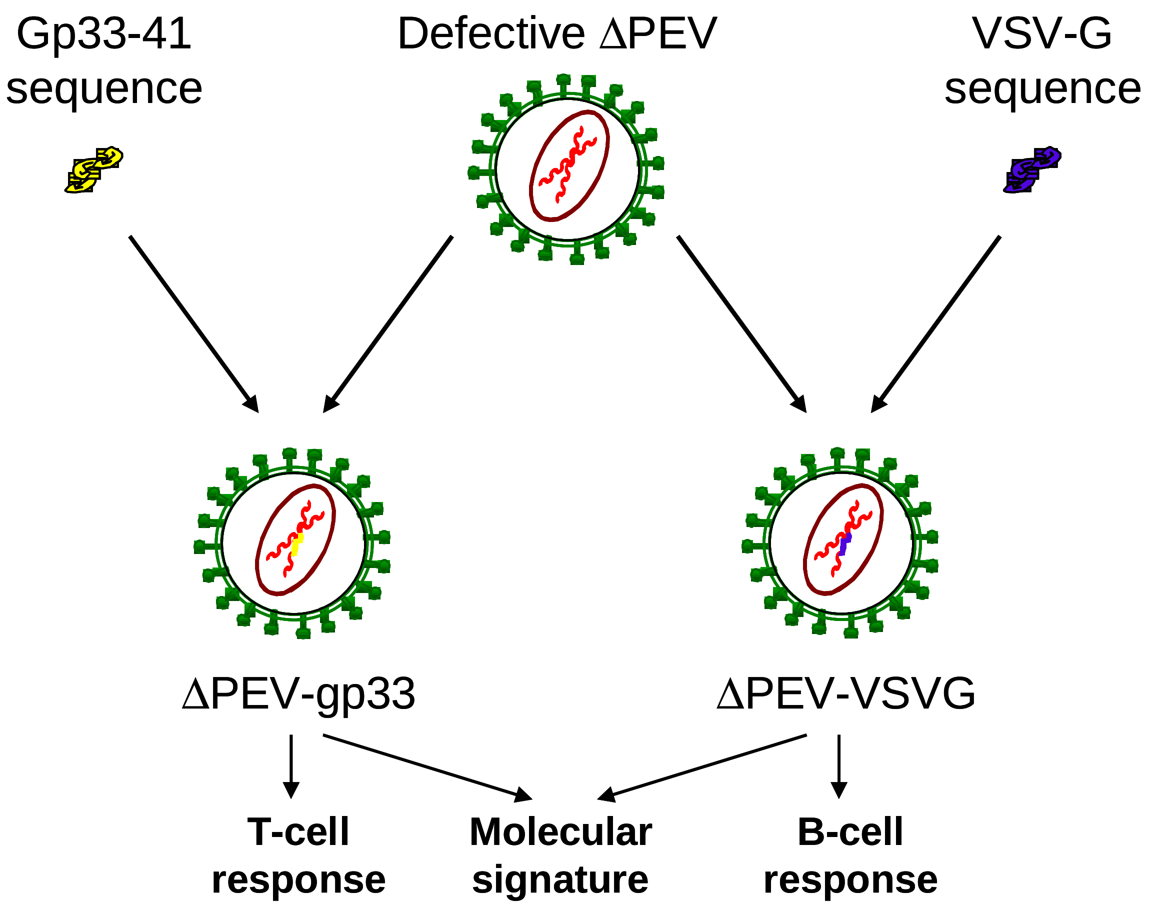

CompuVac's vector construction strategy

A simulated example of GeVaDSs use (4)

- Paul searches for “genetic vaccine standardization” and reads about the CompuVac’s strategy and GeVaDSs database interface.
- Paul contacts CompuVac’s coordinator and receives a login to GeVaDSs
- After browsing GeVaDSs user manual and retrieving Compuvac SOPs, Paul decides to proceed and follows CompuVac’s vector testing:

1. Appropriate vector constructs are produced including standard T-cell (LCMV p33-41) and B-cell (VSV-G protein) antigens.
2. The three vector constructs (PEVdelta, PEVdelta-gp33, PEVdelta-VSVG) are added to GeVaDSs vector database.
3. Mice are immunized with vector constructs according to fully documented and standardized immunization protocols. Considering the natural mucosal infection route of PEV, Paul decides to immunize mice intranasally (IN) with 10^8 virus particles.
4. The immunization protocols are added to GeVaDSs.
5. Experimental vaccines are evaluated against controls (naïve animals) and CompuVac reference vector (internal standard).
6. T-cell & B-cell data as well as molecular signature data can be collected and automatically entered in GeVaDSs database (see Exp. E140, E141, E225, E142, E227). Experimental groups are linked to the corresponding immunization protocols.
7. Experiment reports can be generated for vector performance evaluation.

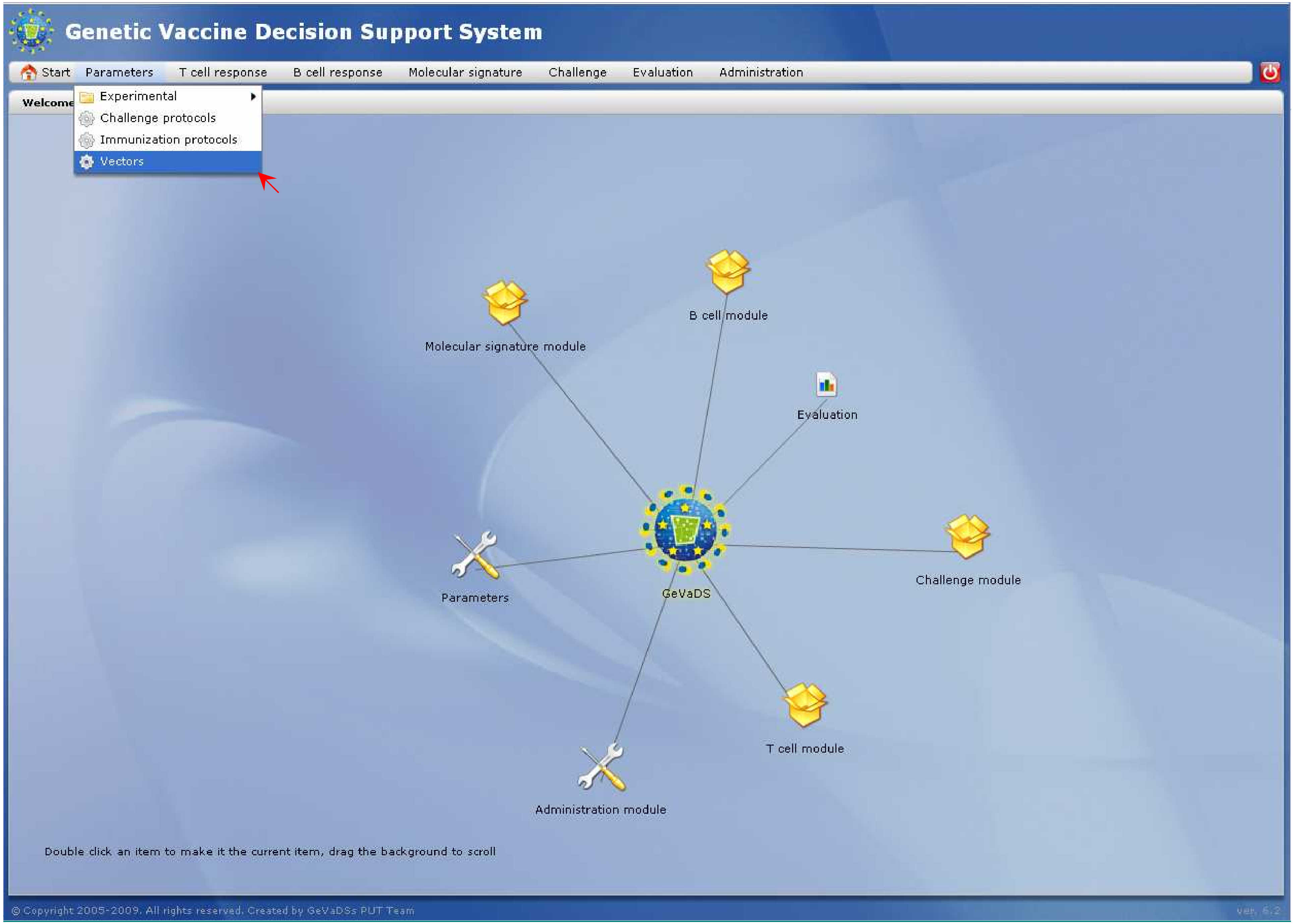
Genetic Vaccine Decision Support System
Start
Parameters
T cell response
B cell response
Molecular signature
Challenge
Evaluation
Administration
Welcome
Experimental
Challenge protocols
Immunization protocols
Vectors
B cell module
Molecular signature module
Evaluation
Parameters
GeVaDS
Challenge module
T cell module
Administration module
Double click an item to make it the current item, drag the background to scroll
© Copyright 2005-2009. All rights reserved. Created by GeVaDSs PUT Team
ver. 6.2

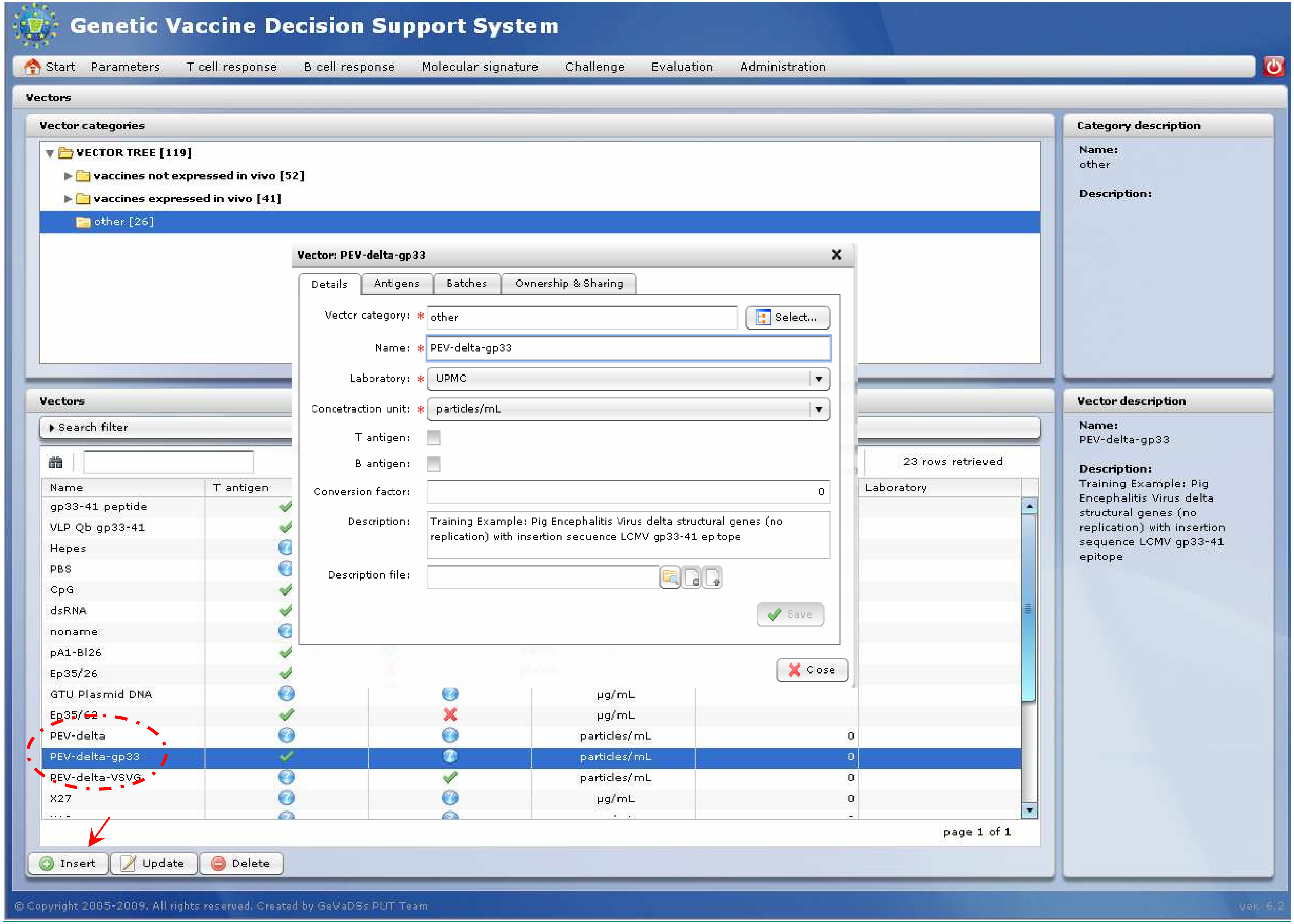
Genetic Vaccine Decision Support System
Start
Parameters
T cell response
B cell response
Molecular signature
Challenge
Evaluation
Administration
Vectors
Vector categories
VECTOR TREE [119]
vaccines not expressed in vivo [52]
vaccines expressed in vivo [41]
other [26]
Category description
Name:
other
Description:
Vector: PEV-delta-gp33
Details
Antigens
Batches
Ownership & Sharing
Vector category: *
other
Select...
Name: *
PEV-delta-gp33
Laboratory: *
UPMC
Concetraction unit: *
particles/mL
T antigen:
B antigen:
Conversion factor:
0
Description:
Training Example: Pig Encephalitis Virus delta structural genes (no replication) with insertion sequence LCMV gp33-41 epitope
Description file:
Save
Close
Vectors
Search filter
23 rows retrieved
Name
T antigen
Laboratory
gp33-41 peptide
VLP Qb gp33-41
Hepes
PBS
CpG
dsRNA
noname
pA1-Bl26
Ep35/26
GTU Plasmid DNA
Ep35/68
PEV-delta
PEV-delta-gp33
PEV-delta-VSVG
X27
µg/mL
particles/mL
page 1 of 1
Insert
Update
Delete
Vector description
Name:
PEV-delta-gp33
Description:
Training Example: Pig Encephalitis Virus delta structural genes (no replication) with insertion sequence LCMV gp33-41 epitope
© Copyright 2005-2009. All rights reserved. Created by GeVaDSs PUT Team
ver. 6.2

A simulated example of GeVaDSs use (5)

- Paul searches for "genetic vaccine standardization" and reads about the CompuVac's strategy and GeVaDSs database interface.
- Paul contacts CompuVac's coordinator and receives a login to GeVaDSs
- After browsing GeVaDSs user manual and retrieving Compuvac SOPs, Paul decides to proceed and follows CompuVac's vector testing:

1. Appropriate vector constructs are produced including standard T-cell (LCMV p33-41) and B-cell (VSV-G protein) antigens.
2. The three vector constructs (PEVdelta, PEVdelta-gp33, PEVdelta-VSVG) are added to GeVaDSs vector database.
3. Mice are immunized with vector constructs according to fully documented and standardized immunization protocols. Considering the natural mucosal infection route of PEV, Paul decides to immunize mice intranasally (IN) with 10^8 virus particles.
4. The immunization protocols are added to GeVaDSs.
5. Experimental vaccines are evaluated against controls (naïve animals) and CompuVac reference vector (internal standard).
6. T-cell & B-cell data as well as molecular signature data can be collected and automatically entered in GeVaDSs database (see Exp. E140, E141, E225, E142, E227). Experimental groups are linked to the corresponding immunization protocols.
7. Experiment reports can be generated for vector performance evaluation.

A simulated example of GeVaDSs use (6)

- Paul searches for “genetic vaccine standardization” and reads about the CompuVac’s strategy and GeVaDSs database interface.
- Paul contacts CompuVac’s coordinator and receives a login to GeVaDSs
- After browsing GeVaDSs user manual and retrieving Compuvac SOPs, Paul decides to proceed and follows CompuVac’s vector testing:

1. Appropriate vector constructs are produced including standard T-cell (LCMV p33-41) and B-cell (VSV-G protein) antigens.
2. The three vector constructs (PEVdelta, PEVdelta-gp33, PEVdelta-VSVG) are added to GeVaDSs vector database.
3. Mice are immunized with vector constructs according to fully documented and standardized immunization protocols. Considering the natural mucosal infection route of PEV, Paul decides to immunize mice intranasally (IN) with 10^8 virus particles.
4. The immunization protocols are added to GeVaDSs.
5. Experimental vaccines are evaluated against controls (naïve animals) and CompuVac reference vector (internal standard).
6. T-cell & B-cell data as well as molecular signature data can be collected and automatically entered in GeVaDSs database (see Exp. E140, E141, E225, E142, E227). Experimental groups are linked to the corresponding immunization protocols.
7. Experiment reports can be generated for vector performance evaluation.

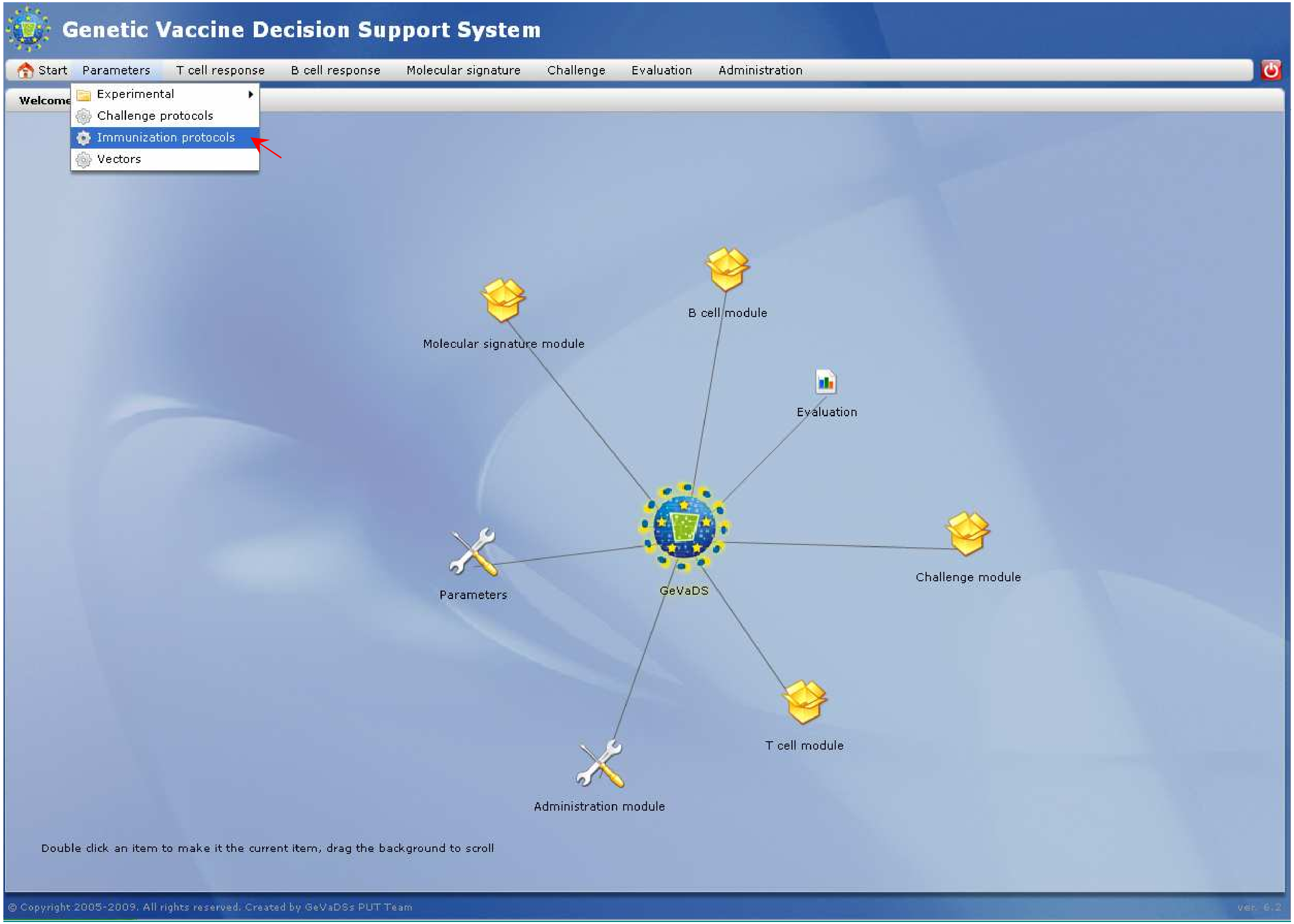
Genetic Vaccine Decision Support System
Start
Parameters
T cell response
B cell response
Molecular signature
Challenge
Evaluation
Administration
Welcome
Experimental
Challenge protocols
Immunization protocols
Vectors
B cell module
Molecular signature module
Evaluation
GeVaDS
Parameters
Challenge module
T cell module
Administration module
Double click an item to make it the current item, drag the background to scroll
© Copyright 2005-2009. All rights reserved. Created by GeVaDSs PUT Team
ver. 6.2

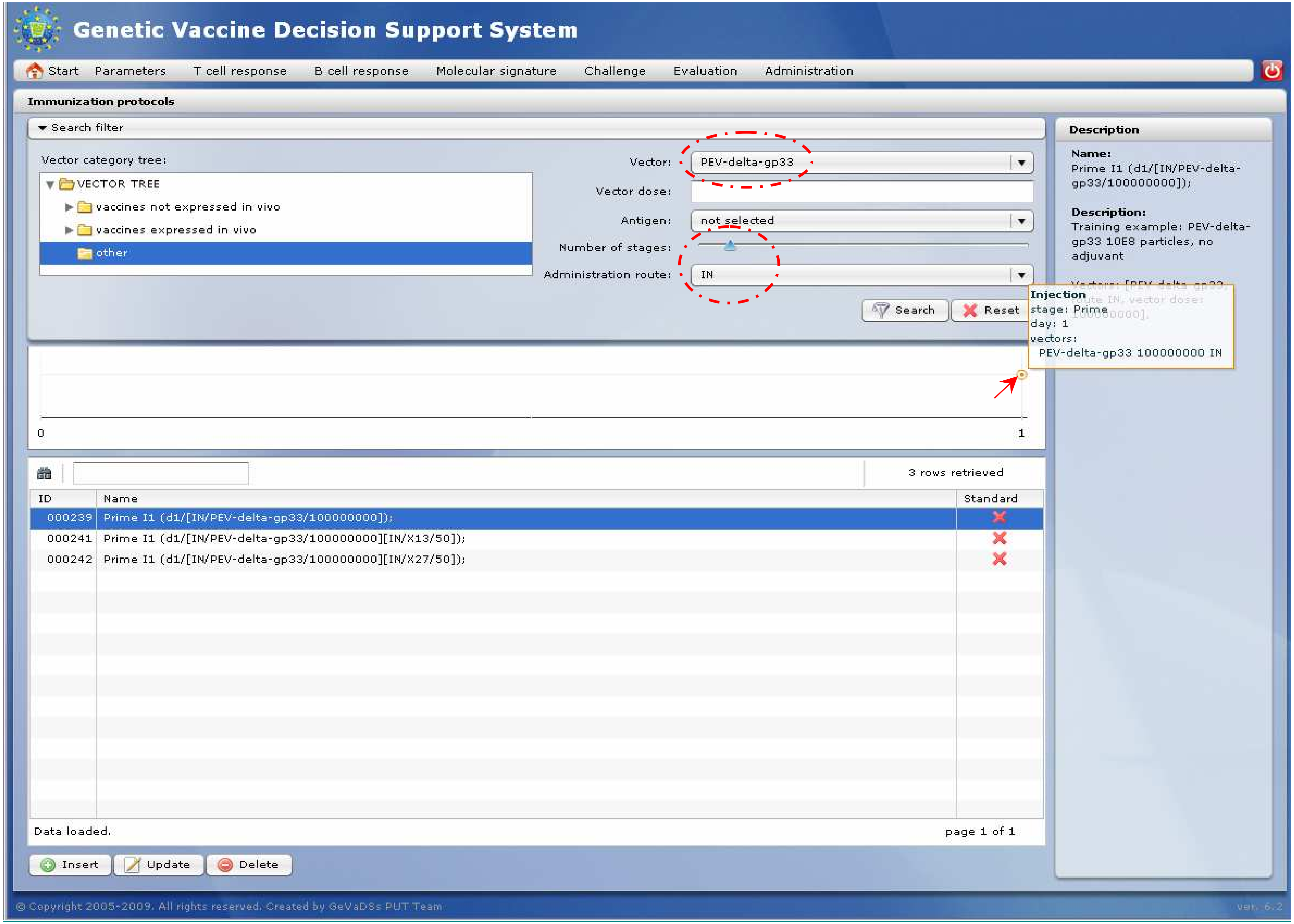
Genetic Vaccine Decision Support System
Start
Parameters
T cell response
B cell response
Molecular signature
Challenge
Evaluation
Administration
Immunization protocols
Search filter
Vector category tree:
VECTOR TREE
vaccines not expressed in vivo
vaccines expressed in vivo
other
Vector:
PEV-delta-gp33
Vector dose:
Antigen:
not selected
Number of stages:
Administration route:
IN
Search
Reset
Injection
stage: Prime
day: 1
vectors:
PEV-delta-gp33 100000000 IN
0
1
3 rows retrieved
ID
Name
Standard
000239 Prime I1 (d1/[IN/PEV-delta-gp33/100000000]);
000241 Prime I1 (d1/[IN/PEV-delta-gp33/100000000][IN/X13/50]);
000242 Prime I1 (d1/[IN/PEV-delta-gp33/100000000][IN/X27/50]);
Data loaded.
page 1 of 1
Insert
Update
Delete
Description
Name:
Prime I1 (d1/[IN/PEV-delta-gp33/100000000]);
Description:
Training example: PEV-delta-gp33 10E8 particles, no adjuvant
© Copyright 2005-2009. All rights reserved. Created by GeVaDSs PUT Team
ver. 6.2

A simulated example of GeVaDSs use (7)

- Paul searches for “genetic vaccine standardization” and reads about the CompuVac’s strategy and GeVaDSs database interface.
- Paul contacts CompuVac’s coordinator and receives a login to GeVaDSs
- After browsing GeVaDSs user manual and retrieving Compuvac SOPs, Paul decides to proceed and follows CompuVac’s vector testing:

1. Appropriate vector constructs are produced including standard T-cell (LCMV p33-41) and B-cell (VSV-G protein) antigens.
2. The three vector constructs (PEVdelta, PEVdelta-gp33, PEVdelta-VSVG) are added to GeVaDSs vector database.
3. Mice are immunized with vector constructs according to fully documented and standardized immunization protocols. Considering the natural mucosal infection route of PEV, Paul decides to immunize mice intranasally (IN) with 10^8 virus particles.
4. The immunization protocols are added to GeVaDSs.
5. Experimental vaccines are evaluated against controls (naïve animals) and CompuVac reference vector (internal standard).
6. T-cell & B-cell data as well as molecular signature data can be collected and automatically entered in GeVaDSs database (see Exp. E140, E141, E225, E142, E227). Experimental groups are linked to the corresponding immunization protocols.
7. Experiment reports can be generated for vector performance evaluation.

A simulated example of GeVaDSs use (8)

- Paul searches for “genetic vaccine standardization” and reads about the CompuVac’s strategy and GeVaDSs database interface.
- Paul contacts CompuVac’s coordinator and receives a login to GeVaDSs
- After browsing GeVaDSs user manual and retrieving Compuvac SOPs, Paul decides to proceed and follows CompuVac’s vector testing:

1. Appropriate vector constructs are produced including standard T-cell (LCMV p33-41) and B-cell (VSV-G protein) antigens.
2. The three vector constructs (PEVdelta, PEVdelta-gp33, PEVdelta-VSVG) are added to GeVaDSs vector database.
3. Mice are immunized with vector constructs according to fully documented and standardized immunization protocols. Considering the natural mucosal infection route of PEV, Paul decides to immunize mice intranasally (IN) with 10^8 virus particles.
4. The immunization protocols are added to GeVaDSs.
5. Experimental vaccines are evaluated against controls (naïve animals) and CompuVac reference vector (internal standard).
6. T-cell & B-cell data as well as molecular signature data can be collected and automatically entered in GeVaDSs database (see Exp. E140, E141, E225, E142, E227). Experimental groups are linked to the corresponding immunization protocols.
7. Experiment reports can be generated for vector performance evaluation.

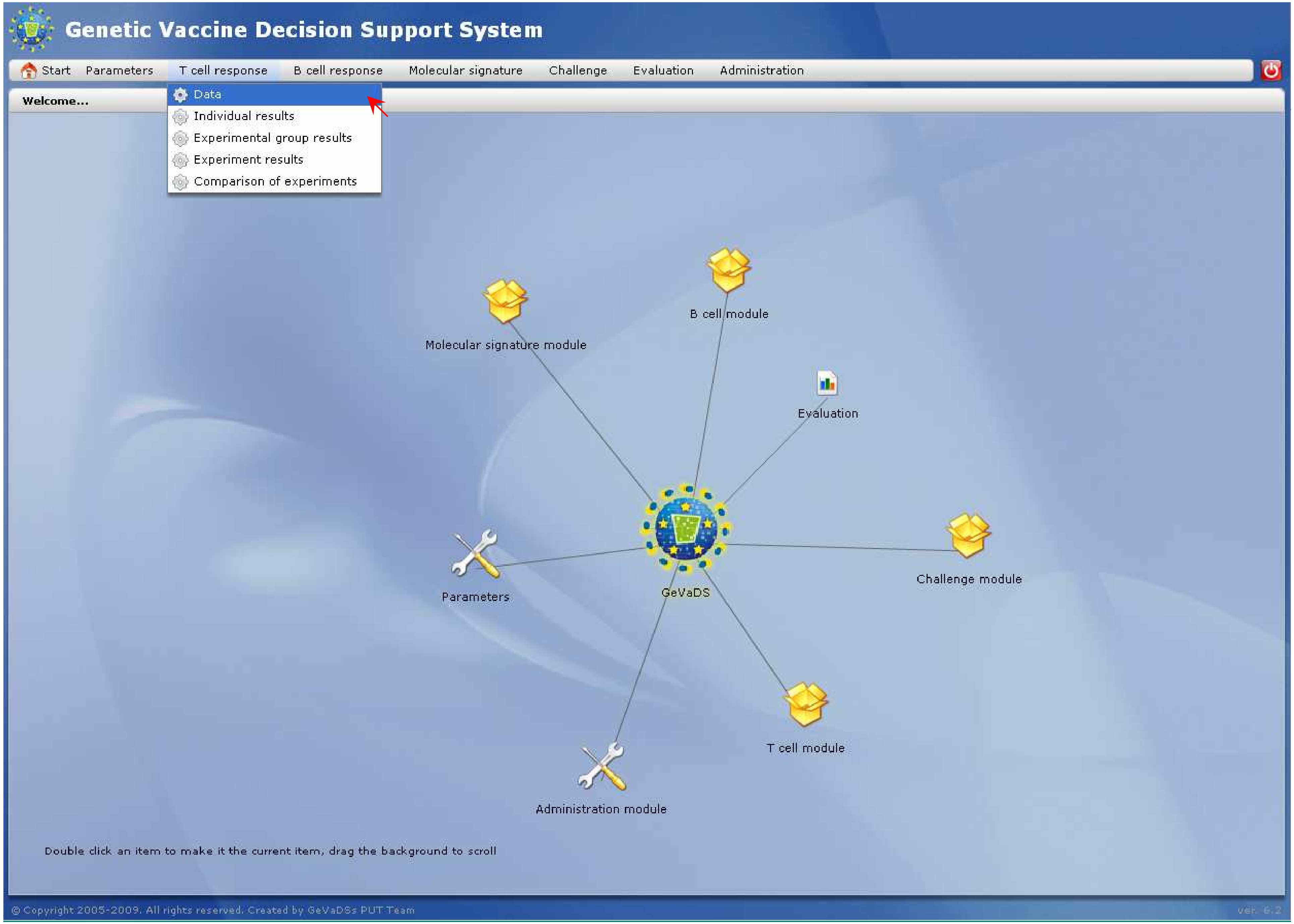
Genetic Vaccine Decision Support System
Start
Parameters
T cell response
B cell response
Molecular signature
Challenge
Evaluation
Administration
Welcome...
Data
Individual results
Experimental group results
Experiment results
Comparison of experiments
B cell module
Molecular signature module
Evaluation
Parameters
GeVaDS
Challenge module
T cell module
Administration module
Double click an item to make it the current item, drag the background to scroll
© Copyright 2005-2009. All rights reserved. Created by GeVaDSs PUT Team
ver. 6.2

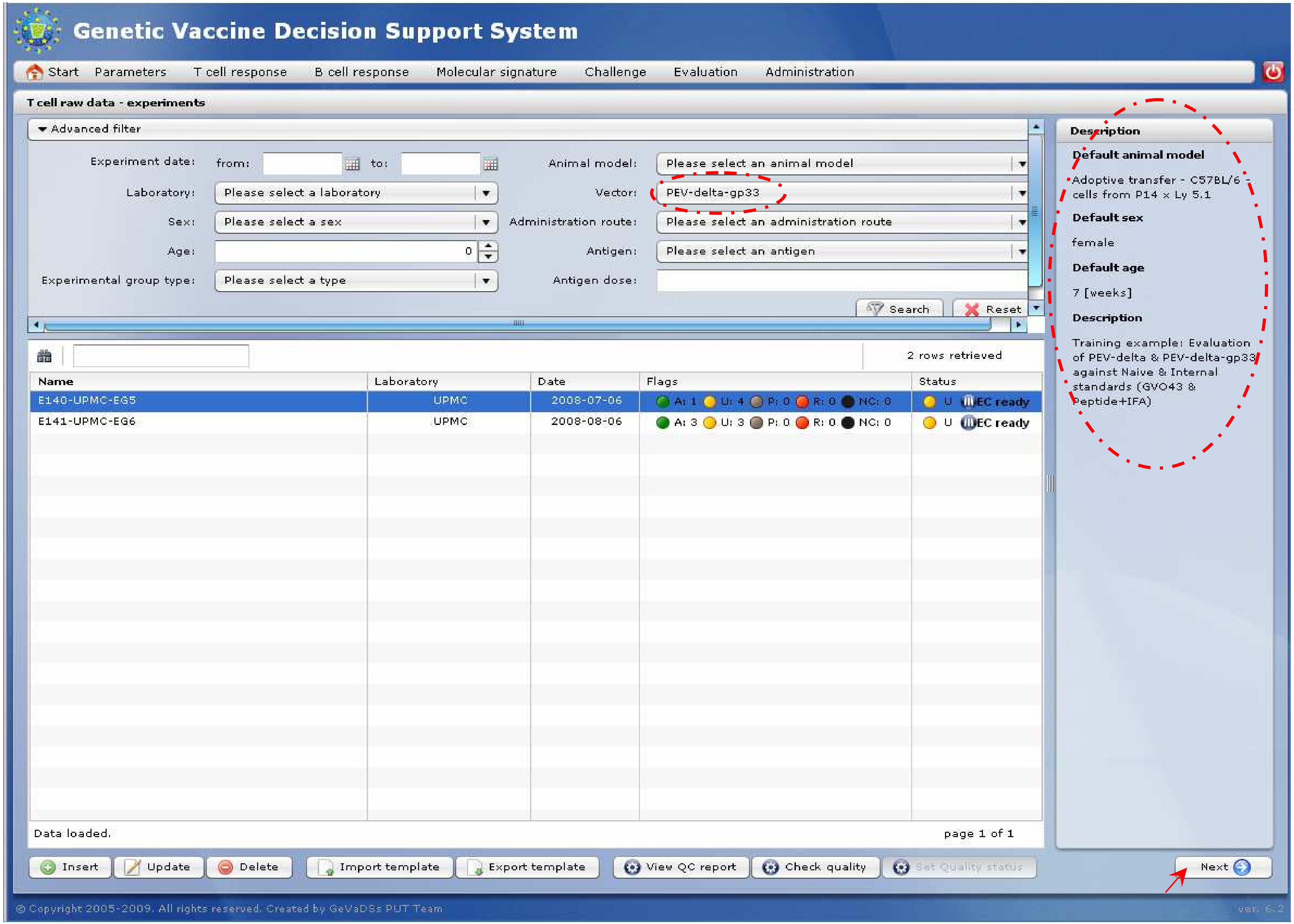
Genetic Vaccine Decision Support System
Start
Parameters
T cell response
B cell response
Molecular signature
Challenge
Evaluation
Administration
T cell raw data - experiments
Advanced filter
Experiment date: from: to:
Laboratory: Please select a laboratory
Sex: Please select a sex
Age: 0
Experimental group type: Please select a type
Animal model: Please select an animal model
Vector: PEV-delta-gp33
Administration route: Please select an administration route
Antigen: Please select an antigen
Antigen dose:
Search
Reset
2 rows retrieved
Name
Laboratory
Date
Flags
Status
E140-UPMC-EG5
UPMC
2008-07-06
A: 1 U: 4 P: 0 R: 0 NC: 0
U EC ready
E141-UPMC-EG6
UPMC
2008-08-06
A: 3 U: 3 P: 0 R: 0 NC: 0
U EC ready
Data loaded.
page 1 of 1
Description
Default animal model
Adoptive transfer - C57BL/6 - cells from P14 x Ly 5.1
Default sex
female
Default age
7 [weeks]
Description
Training example: Evaluation of PEV-delta & PEV-delta-gp33 against Naive & Internal standards (GVO43 & Peptide+IFA)
Insert
Update
Delete
Import template
Export template
View QC report
Check quality
Set Quality status
Next
© Copyright 2005-2009. All rights reserved. Created by GeVaDSs PUT Team
ver. 6.2

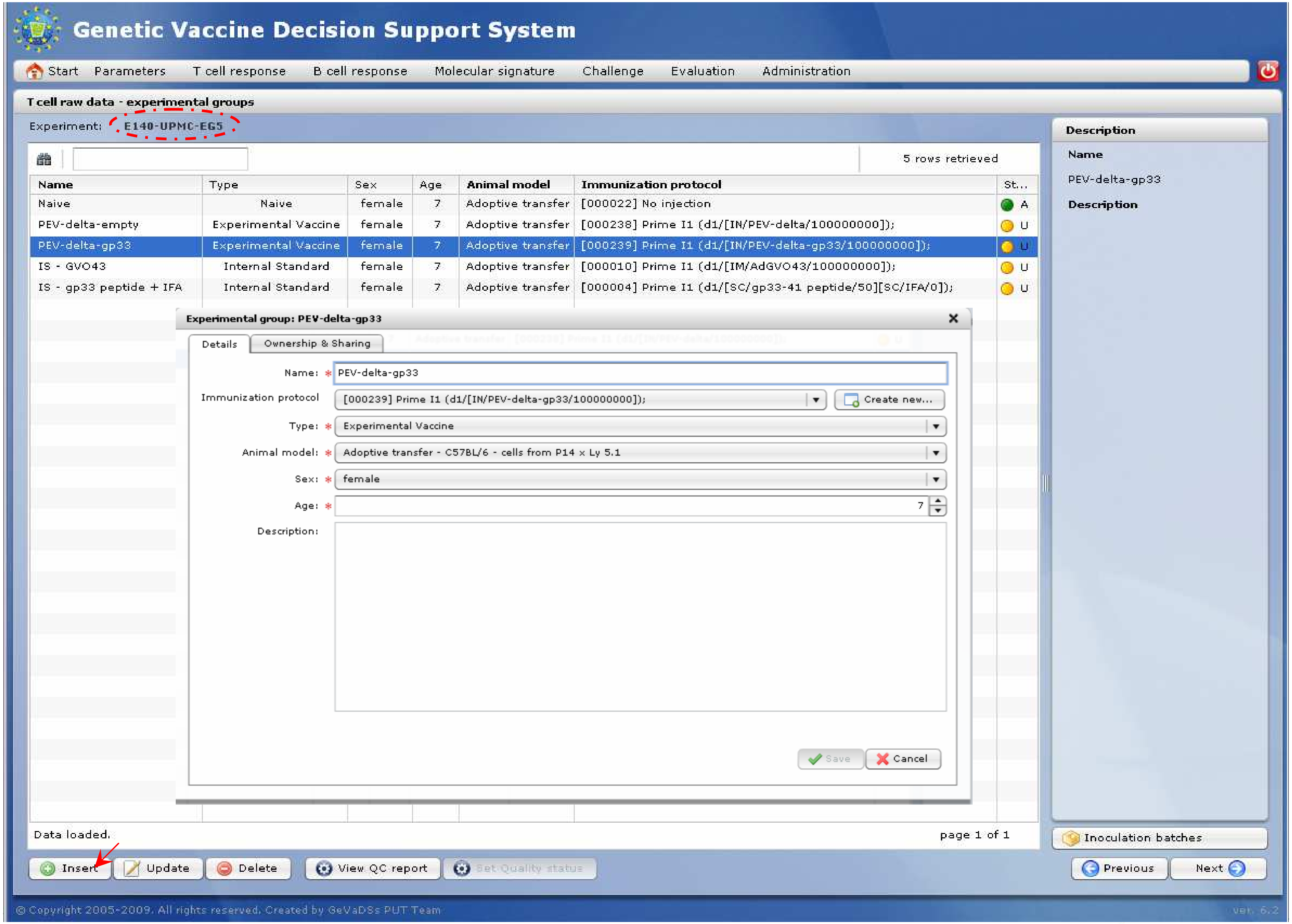
Genetic Vaccine Decision Support System
Start
Parameters
T cell response
B cell response
Molecular signature
Challenge
Evaluation
Administration
T cell raw data - experimental groups
Experiment: E140-UPMC-EG5
5 rows retrieved
Name
Type
Sex
Age
Animal model
Immunization protocol
St...
Naive | Naive | female | 7 | Adoptive transfer | [000022] No injection | A
PEV-delta-empty | Experimental Vaccine | female | 7 | Adoptive transfer | [000238] Prime I1 (d1/[IN/PEV-delta/100000000]); | U
PEV-delta-gp33 | Experimental Vaccine | female | 7 | Adoptive transfer | [000239] Prime I1 (d1/[IN/PEV-delta-gp33/100000000]); | U
IS - GVO43 | Internal Standard | female | 7 | Adoptive transfer | [000010] Prime I1 (d1/[IM/AdGVO43/100000000]); | U
IS - gp33 peptide + IFA | Internal Standard | female | 7 | Adoptive transfer | [000004] Prime I1 (d1/[SC/gp33-41 peptide/50][SC/IFA/0]); | U
Experimental group: PEV-delta-gp33
Details
Ownership & Sharing
Name: * PEV-delta-gp33
Immunization protocol [000239] Prime I1 (d1/[IN/PEV-delta-gp33/100000000]);
Create new...
Type: * Experimental Vaccine
Animal model: * Adoptive transfer - C57BL/6 - cells from P14 x Ly 5.1
Sex: * female
Age: * 7
Description:
Save
Cancel
Description
Name
PEV-delta-gp33
Description
Data loaded.
page 1 of 1
Inoculation batches
Insert
Update
Delete
View QC report
Set Quality status
Previous
Next
© Copyright 2005-2009. All rights reserved. Created by GeVaDSs PUT Team
ver. 6.2

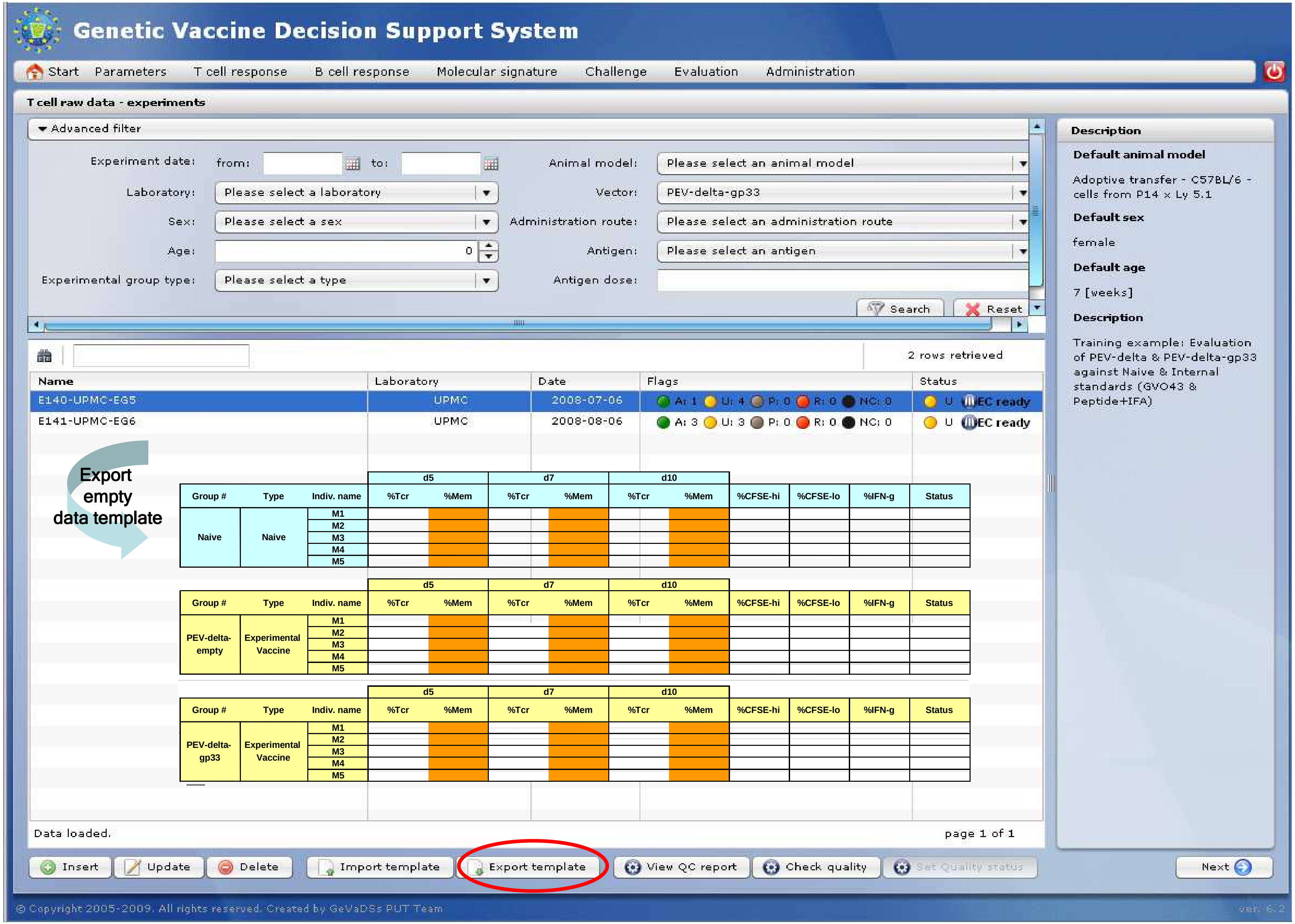

			d5		d7		d10					
Group #	Type	Indiv. name	%Tcr	%Mem	%Tcr	%Mem	%Tcr	%Mem	%CFSE-hi	%CFSE-lo	%IFN-g	Status
Naive	Naive	M1										
		M2										
		M3										
		M4										
		M5										

			d5		d7		d10					
Group #	Type	Indiv. name	%Tcr	%Mem	%Tcr	%Mem	%Tcr	%Mem	%CFSE-hi	%CFSE-lo	%IFN-g	Status
PEV-delta-empty	Experimental Vaccine	M1										
		M2										
		M3										
		M4										
		M5										

			d5		d7		d10					
Group #	Type	Indiv. name	%Tcr	%Mem	%Tcr	%Mem	%Tcr	%Mem	%CFSE-hi	%CFSE-lo	%IFN-g	Status
PEV-delta-gp33	Experimental Vaccine	M1										
		M2										
		M3										
		M4										
		M5										

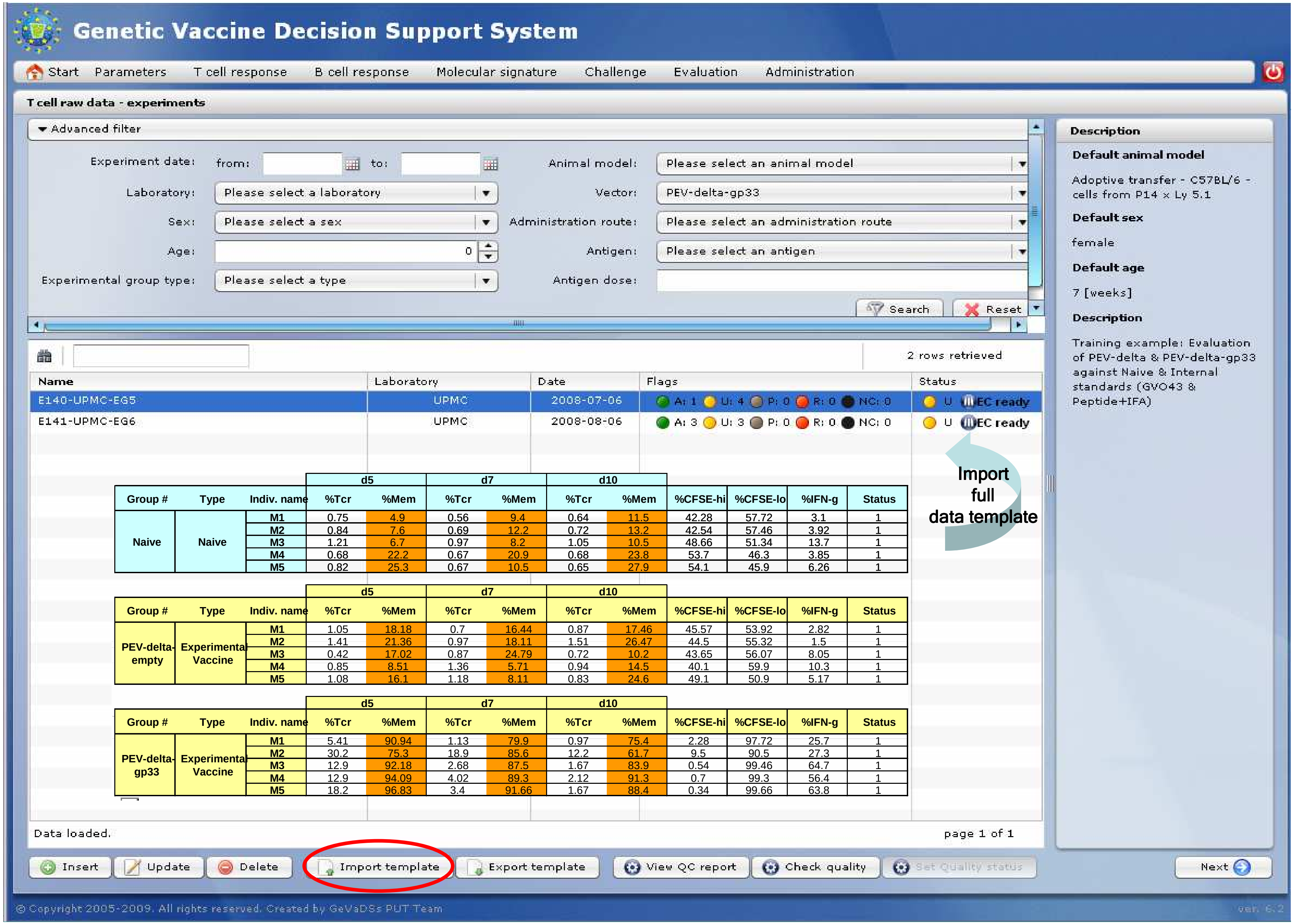

			d5		d7		d10					
Group #	Type	Indiv. name	%Tcr	%Mem	%Tcr	%Mem	%Tcr	%Mem	%CFSE-hi	%CFSE-lo	%IFN-g	Status
Naive	Naive	M1	0.75	4.9	0.56	9.4	0.64	11.5	42.28	57.72	3.1	1
		M2	0.84	7.6	0.69	12.2	0.72	13.2	42.54	57.46	3.92	1
		M3	1.21	6.7	0.97	8.2	1.05	10.5	48.66	51.34	13.7	1
		M4	0.68	22.2	0.67	20.9	0.68	23.8	53.7	46.3	3.85	1
		M5	0.82	25.3	0.67	10.5	0.65	27.9	54.1	45.9	6.26	1

			d5		d7		d10					
Group #	Type	Indiv. name	%Tcr	%Mem	%Tcr	%Mem	%Tcr	%Mem	%CFSE-hi	%CFSE-lo	%IFN-g	Status
PEV-delta-empty	Experimental Vaccine	M1	1.05	18.18	0.7	16.44	0.87	17.46	45.57	53.92	2.82	1
		M2	1.41	21.36	0.97	18.11	1.51	26.47	44.5	55.32	1.5	1
		M3	0.42	17.02	0.87	24.79	0.72	10.2	43.65	56.07	8.05	1
		M4	0.85	8.51	1.36	5.71	0.94	14.5	40.1	59.9	10.3	1
		M5	1.08	16.1	1.18	8.11	0.83	24.6	49.1	50.9	5.17	1

			d5		d7		d10					
Group #	Type	Indiv. name	%Tcr	%Mem	%Tcr	%Mem	%Tcr	%Mem	%CFSE-hi	%CFSE-lo	%IFN-g	Status
PEV-delta-gp33	Experimental Vaccine	M1	5.41	90.94	1.13	79.9	0.97	75.4	2.28	97.72	25.7	1
		M2	30.2	75.3	18.9	85.6	12.2	61.7	9.5	90.5	27.3	1
		M3	12.9	92.18	2.68	87.5	1.67	83.9	0.54	99.46	64.7	1
		M4	12.9	94.09	4.02	89.3	2.12	91.3	0.7	99.3	56.4	1
		M5	18.2	96.83	3.4	91.66	1.67	88.4	0.34	99.66	63.8	1

A simulated example of GeVaDSs use (9)

- Paul searches for “genetic vaccine standardization” and reads about the CompuVac’s strategy and GeVaDSs database interface.
- Paul contacts CompuVac’s coordinator and receives a login to GeVaDSs
- After browsing GeVaDSs user manual and retrieving Compuvac SOPs, Paul decides to proceed and follows CompuVac’s vector testing:

1. Appropriate vector constructs are produced including standard T-cell (LCMV p33-41) and B-cell (VSV-G protein) antigens.
2. The three vector constructs (PEVdelta, PEVdelta-gp33, PEVdelta-VSVG) are added to GeVaDSs vector database.
3. Mice are immunized with vector constructs according to fully documented and standardized immunization protocols. Considering the natural mucosal infection route of PEV, Paul decides to immunize mice intranasally (IN) with 10^8 virus particles.
4. The immunization protocols are added to GeVaDSs.
5. Experimental vaccines are evaluated against controls (naïve animals) and CompuVac reference vector (internal standard).
6. T-cell & B-cell data as well as molecular signature data can be collected and automatically entered in GeVaDSs database (see Exp. E140, E141, E225, E142, E227). Experimental groups are linked to the corresponding immunization protocols.
7. Experiment reports can be generated for vector performance evaluation.

A simulated example of GeVaDSs use (10)

- Standardized comparison of ΔPEV-gp33 with other GeVaDSs vaccines:

T-cell data have been normalized against the GVO43 Internal Standard (=1) across experiments.

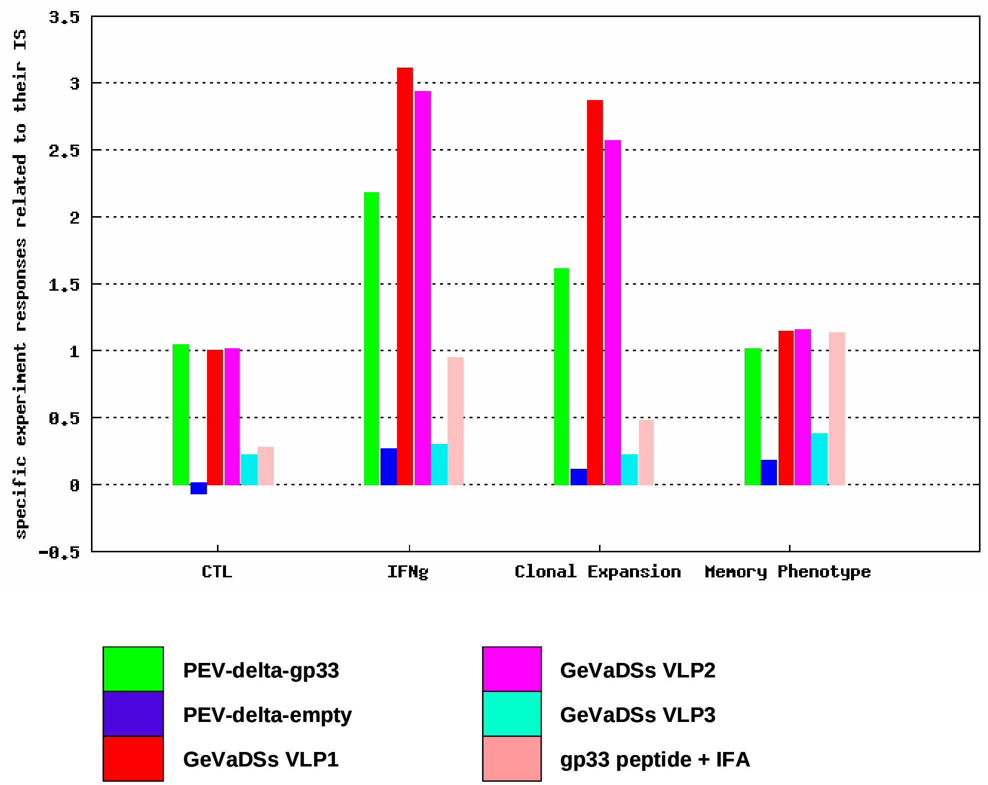

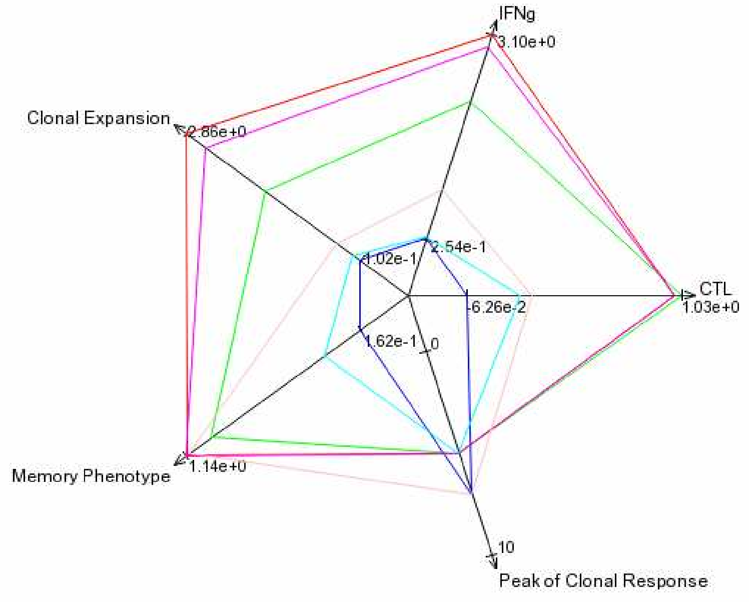

from GeVaDSs Exp. E140 vs. E225

© Compuvac, 2009

→ΔPEV-gp33 performance is in-between that of the GVO43 internal positive control and GeVaDSs' validated "best" T-cell vaccines.

A simulated example of GeVaDSs use (11)

- Paul goes on to check for PEV’s capacity to produce antibodies
- GeVaDSs B-cell experiment → no significant neutralizing Ab produced against the VSV-G model antigen (see Exp. E142)
- Paul then tests PEV-induced Ab production in the presence of adjuvants.
- Two experimental adjuvants (X13 & X27) are tested, with the following questions:
  - Is T-cell response against PEV-gp33 altered?
  - Can Ab produced by B-cell response neutralize PEV-VSVG ?
  - What impact do adjuvants have on “toxicity” signatures of dendritic cells?

A simulated example of GeVaDSs use (12)

- Is T-cell response against PEV-gp33 altered?

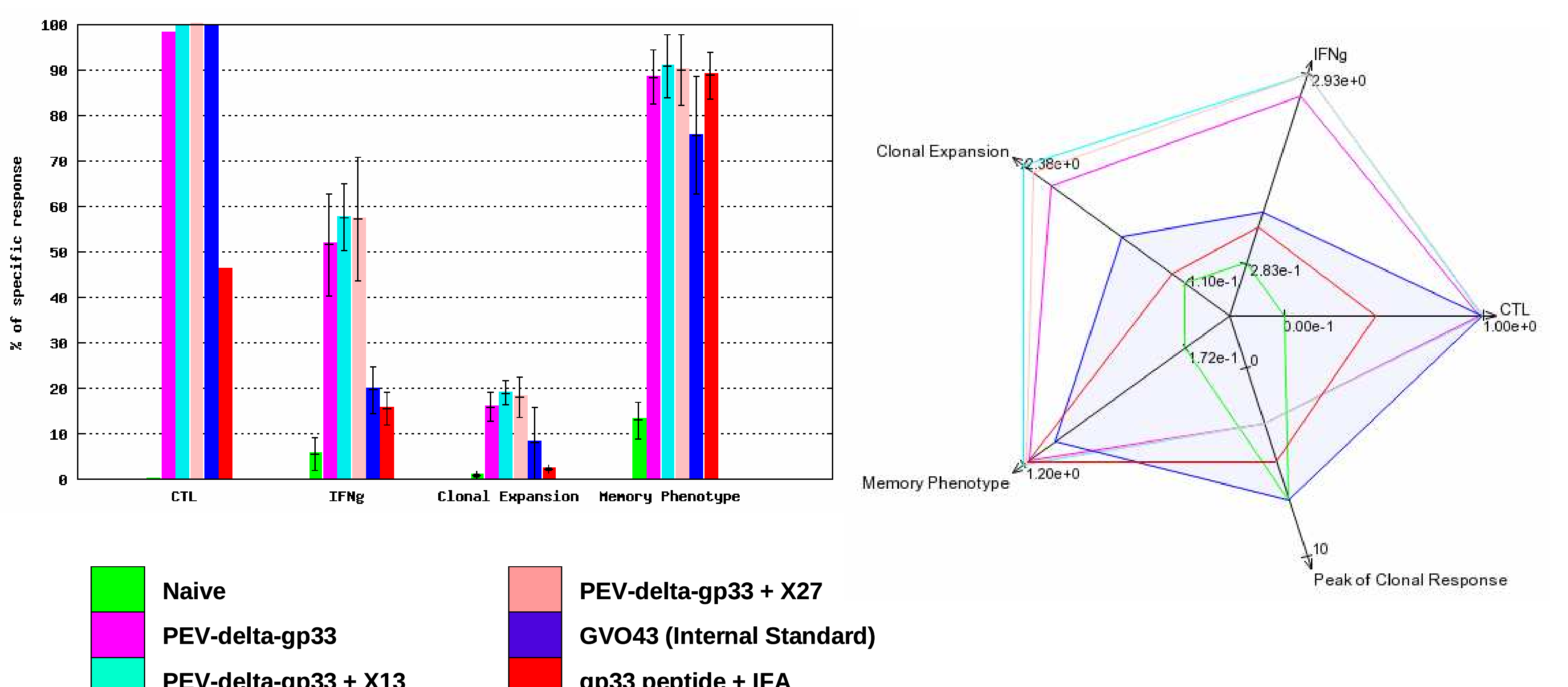

from GeVaDSs Exp. E141

© Compuvac, 2009

→ΔPEV-gp33 performs similarly in the presence of X13/X27 adjuvants.

A simulated example of GeVaDSs use (13)

- Can Ab produced by B-cell response neutralize PEV-VSVG ?

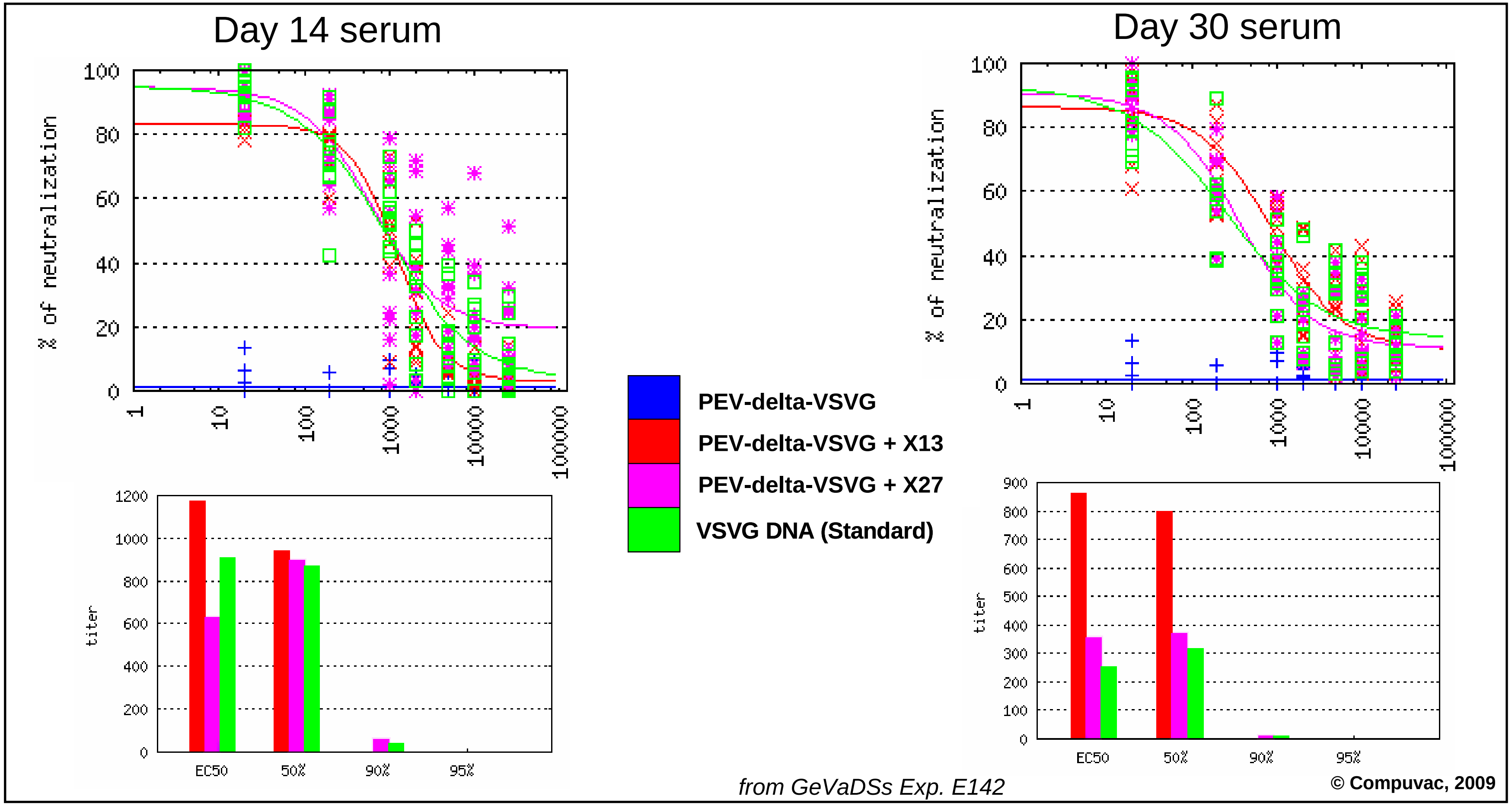

→ΔPEV-VSVG with X13/X27 adjuvants elicits anti-VSVG neutralizing antibodies.

→X13 adjuvant is more potent that X27.

A simulated example of GeVaDSs use (14)

- What impact do adjuvants have on “toxicity” signatures of dendritic cells?

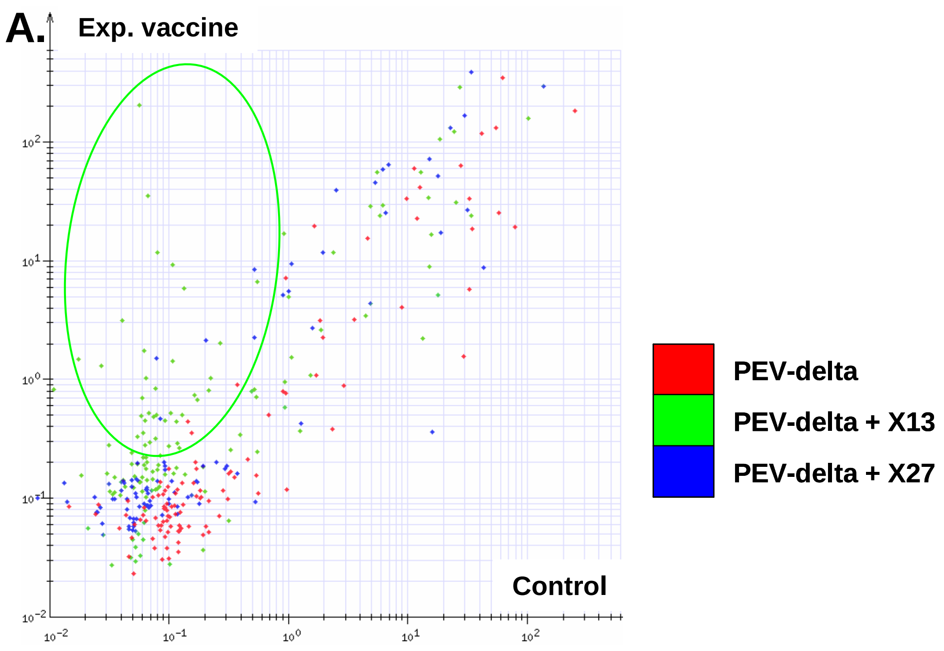

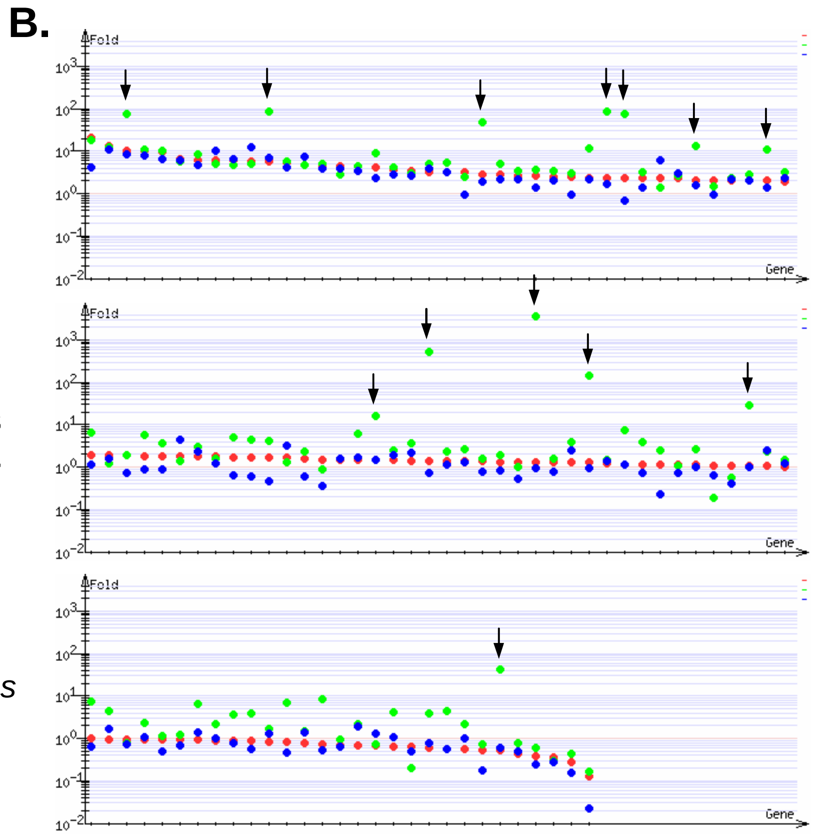

Whole genome microarray data has been filtered out on GeVaDSs “toxicity” gene filter (the level of which should not be altered).

(A) Gene expression level of vaccinated against control groups.
(B) Fold-increase in gene expression level of vaccinated against control groups.

from GeVaDSs Exp. E227

© Compuvac, 2009

→X13 adjuvant significantly alters the toxicity profile of dendritic cells compared to PEV alone or with X27. X13 should preferably not be used.

A simulated example of GeVaDSs use (15)

- After a limited number of experiments, Paul can conclude on the overall potential of PEV as a new vector platform:

Vector combination	T-cell response	B-cell response	Molecular signature
ΔPEV alone	++	-	OK
ΔPEV + X13 adjuvant	++	++	Potential safety issues
ΔPEV + X27 adjuvant	++	+	OK

→ PEV is a good vaccine vector platform candidate, as revealed by GeVaDSs standardized evaluation strategy
