# Supplementary material for: GeVaDSs – decision support system for novel Genetic Vaccine development process
Source: BMC Bioinformatics. 2012 May 10;13:91. doi: 10.1186/1471-2105-13-91 (PMC3531312; doi:10.1186/1471-2105-13-91)
Supplement: Additional file 2 — GeVaDSs workflow. The different steps of the GeVaDSs workflow are summarized in the light of the example provided in Additional file 1. [file 1471-2105-13-91-S2.pdf]

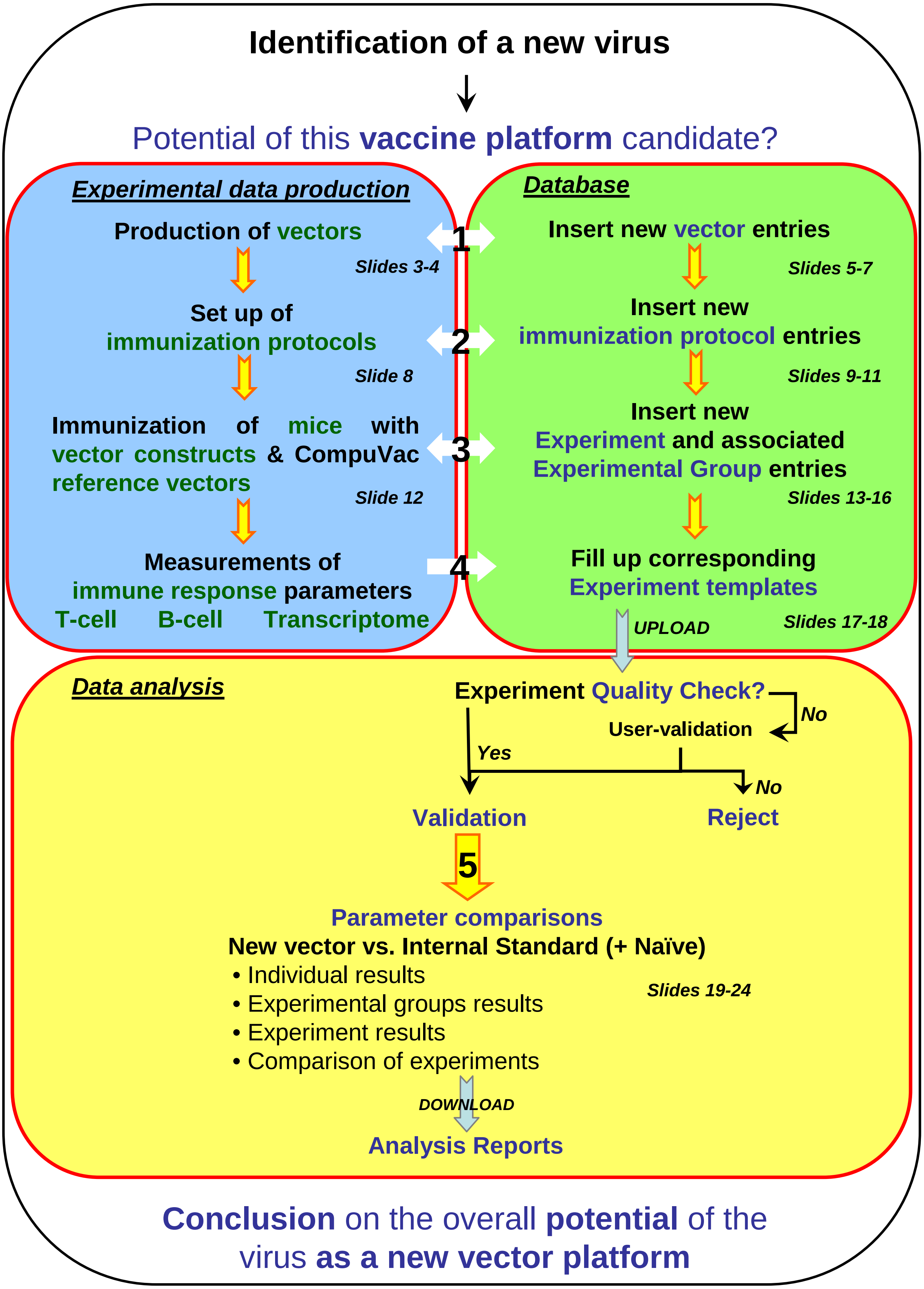
Identification of a new virus
Potential of this vaccine platform candidate?
Experimental data production
Production of vectors
Slides 3-4
Set up of immunization protocols
Slide 8
Immunization of mice with vector constructs & CompuVac reference vectors
Slide 12
Measurements of immune response parameters
T-cell B-cell Transcriptome
1
2
3
4
Database
Insert new vector entries
Slides 5-7
Insert new immunization protocol entries
Slides 9-11
Insert new Experiment and associated Experimental Group entries
Slides 13-16
Fill up corresponding Experiment templates
UPLOAD
Slides 17-18
Data analysis
Experiment Quality Check?
No
User-validation
Yes
No
Validation
Reject
5
Parameter comparisons
New vector vs. Internal Standard (+ Naïve)
• Individual results
• Experimental groups results
• Experiment results
• Comparison of experiments
Slides 19-24
DOWNLOAD
Analysis Reports
Conclusion on the overall potential of the virus as a new vector platform
